# Generation of Photocaged Nanobodies for Intracellular Applications in an Animal Using Genetic Code Expansion and Computationally Guided Protein Engineering**

**DOI:** 10.1002/cbic.202200321

**Published:** 2022-07-07

**Authors:** Jack M. O'Shea, Angeliki Goutou, Jack Brydon, Cyrus R. Sethna, Christopher W. Wood, Sebastian Greiss

**Affiliations:** ^1^ Centre for Discovery Brain Sciences University of Edinburgh, Hugh Robson Building George Square Edinburgh EH8 9XD UK; ^2^ MRC Institute of Genetics & Molecular Medicine University of Edinburgh, Western General Hospital Crewe Road South Edinburgh EH4 2XR UK; ^3^ Institute of Quantitative Biology, Biochemistry and Biotechnology University of Edinburgh, Roger Land Building King's Buildings Edinburgh EH9 3JQ UK

**Keywords:** *C. elegans*, computational alanine scanning, nanobodies, photocaged non-canonical amino acids, protein engineering

## Abstract

Nanobodies are becoming increasingly popular as tools for manipulating and visualising proteins *in vivo*. The ability to control nanobody/antigen interactions using light could provide precise spatiotemporal control over protein function. We develop a general approach to engineer photo‐activatable nanobodies using photocaged amino acids that are introduced into the target binding interface by genetic code expansion. Guided by computational alanine scanning and molecular dynamics simulations, we tune nanobody/target binding affinity to eliminate binding before uncaging. Upon photo‐activation using 365 nm light, binding is restored. We use this approach to generate improved photocaged variants of two anti‐GFP nanobodies that function robustly when directly expressed in a complex intracellular environment together with their antigen. We apply them to control subcellular protein localisation in the nematode worm *Caenorhabditis elegans*. Our approach applies predictions derived from computational modelling directly in a living animal and demonstrates the importance of accounting for *in vivo* effects on protein‐protein interactions.

## Graphical abstract

**Light‐activated nanobodies** for intracellular use were created using a combination of genetic code expansion and computationally guided engineering of the binding interface. The engineered nanobodies allow optical control of subcellular protein localisation in living *Caenorhabditis elegans*.

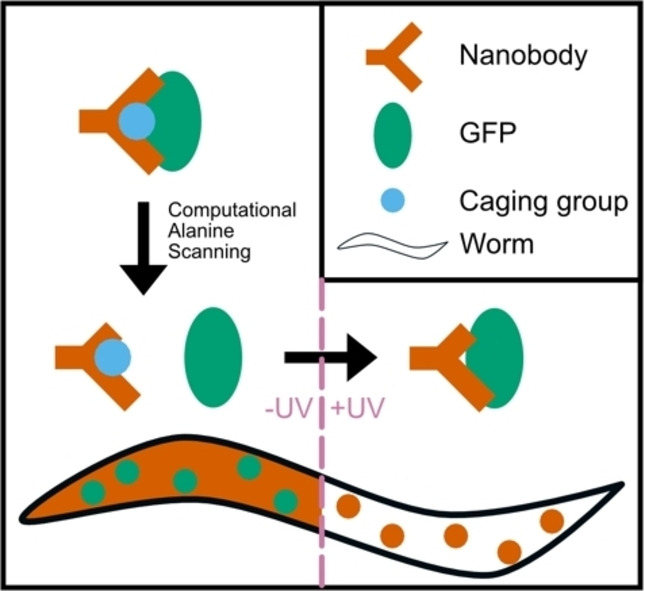

## Introduction

Nanobodies are small, 12–15 kD, fragments of camelid heavy‐chain only antibodies. Despite their small size, which corresponds to around 10 % the size of standard antibodies, they exhibit potent and specific target binding. In recent years, nanobodies have been developed into indispensable tools for investigating protein function, in part due to the fact that they can be easily expressed *in vivo*.[^1^, ^2^] This unique set of properties has been leveraged to develop a variety of tools for labelling and manipulating proteins, to gain insights into biological processes. Fluorescently tagged nanobodies, termed “chromobodies”, have been used for *in vivo* antigen labelling.^[3]^ The ability of nanobodies to bind and interfere with specific protein domains makes them effective and highly specific inhibitors,[^4^, ^5^, ^6^, ^7^, ^8^] capable of blocking pathogenic functions of proteins such as the *Clostridium difficile* toxin CDT,^[9]^ and the SARS‐CoV‐2 Spike.[^10^, ^11^] Besides direct inhibition, nanobodies have been used to investigate antigen function by targeted proteasomal degradation via recruitment of the cellular ubiquitination machinery[^12^, ^13^, ^14^, ^15^, ^16^] or by controlling the antigen‘s subcellular localisation.[^3^, ^4^, ^7^, ^8^, ^17^, ^18^, ^19^, ^20^] The binding specificity of nanobodies and ease of intracellular expression has been exploited to improve ligand affinity to receptors for observation of rare binding events.^[21]^ Additionally, nanobodies have been used to deliver photo‐switchable ligands to receptors for optical control of receptor activation.^[22]^ Their small recognition epitopes even facilitate tools wherein two nanobodies that bind different regions of GFP can control transcription by using GFP as a bridge to bring together split proteins, such as split transcription factors and split Cre recombinase.[^23^, ^24^] Nanobodies have been employed as tools in a wide range of model systems, including mammalian cells, mice, zebrafish, and *Caenorhabditis elegans*.^[2]^

A limitation of native nanobodies is that their binding cannot be modulated after expression. The ability to induce antigen binding would significantly increase the applicability of nanobody‐based tools by adding a level of spatiotemporal control absent when using native nanobodies. Several avenues have been explored to install the ability to control nanobody/antigen interaction. Insertion of a ligand‐binding domain into the loop regions of various nanobodies has been used to impart chemo‐inducibility, making antigen binding dependent on small molecule ligands.^[25]^ However, chemical control of nanobody binding does not offer spatial control and offers only limited temporal control due to slow diffusion kinetics of the required chemical ligand, especially in multicellular systems. Optical control is an attractive alternative to chemical control, since it potentially allows high‐precision spatiotemporal manipulation.

**Figure 1:**
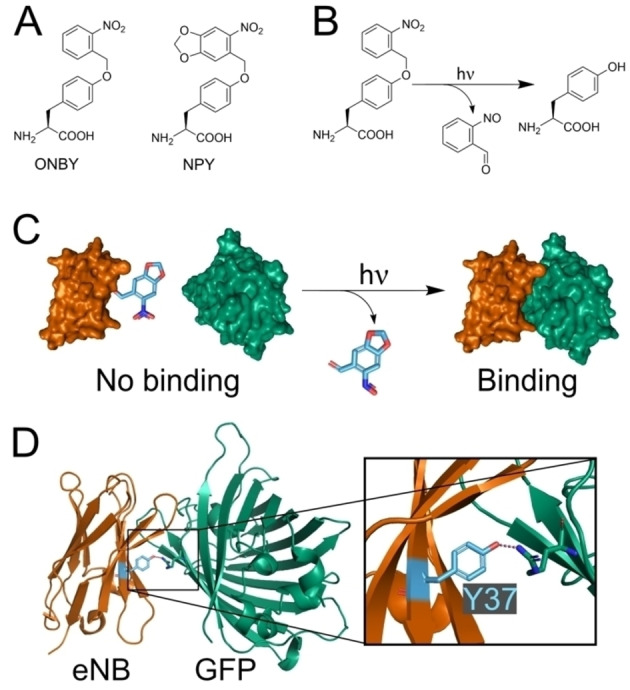
A. Chemical structures of the photocaged tyrosines ortho‐nitrobenzyl tyrosine (ONBY) and nitropiperonyl tyrosine (NPY). B. The uncaging reaction of ONBY. Uncaging of NPY follows the same mechanism. C. Schematic for photoinducible protein‐protein interactions using photocaged amino acids. The photocaging group (in blue) prevents interaction of the binding partners but is removed by illumination. D. Crystal structure (PDB: 3K1K) of the interaction of the anti‐GFP “enhancer” nanobody (eNB) and GFP. Highlighted in blue is Y37 of eNB, which forms a polar interaction with R168 of GFP.

Three avenues to install optical control in nanobodies have been explored. The fusion of split nanobody fragments with photo‐inducible dimerisers allows photoinducible binding, however this approach can be limited by slow kinetics, and binding saturation is only achieved after extended illumination.^[26]^ Insertion of photo‐switchable domains into nanobody loops also achieved photoinducible antigen binding or dissociation, but the direction and magnitude of the effect can be unpredictable.^[27]^ These two approaches also suffer from requiring blue light for induction, which can interfere with imaging and also makes them incompatible with other blue light dependent optogenetic tools.[^16^, ^22^, ^28^] The third approach involves the use of genetic code expansion^[29]^ to incorporate photocaged amino acids (Figure 1A) into the binding interface of the nanobody with its antigen, thereby preventing binding until the caging group is removed by illumination (Figure 1B, 1 C). Uncaging of photocaged amino acids is rapid, can be performed with excellent spatial resolution, and the wavelengths involved are compatible with most imaging and optogenetics techniques.^[30]^

While the genetic code of several multicellular organisms has been expanded to date, including *C. elegans*, Drosophila, mouse, and zebrafish, only very few types of ncAA have been established for use in animals as compared to the repertoire available for mammalian cell culture or bacterial expression systems^[31]^. The use of photocaged tyrosine has until now not been established in any animal model. Tyrosine is an attractive target for photocaging and can be used to control the activity of a number of enzyme classes such as proteases, DNA recombinases, or DNA and RNA polymerases.^[32]^ Furthermore, tyrosine is an important target for post‐translational regulation via phosphorylation.^[33]^

Photocaged tyrosines have been used successfully to reduce nanobody/antigen binding *in vitro* by replacing key tyrosines on nanobodies with their photocaged counterparts.[^32^, ^34^]

Intracellular use of photo‐caged nanobodies was first shown by delivering purified photocaged nanobodies synthesised in *E. coli* to HeLa cells expressing the antigen,^[35]^ and subsequently by directly expressing a photo‐caged nanobody in a cultured human cell line.[^36^, ^37^]

A number of factors influence nanobody/antigen interaction, including pH, ionic concentration, incubation time, and concentration of nanobody and antigen.^[38]^ The disruption of binding by the photocaged amino acid observed in one system may therefore not correctly predict binding when applying photocaged nanobodies in other systems or at different concentrations. The difference between the encountered conditions could result in failure of the photocaged amino acid to abolish the nanobody/antigen interaction *in vivo*. In this case, a solution would be the modification of the binding interface through the introduction of further mutations to tune the binding strength for the desired environment.

Here we show that the introduction of a photo‐caging group into intracellularly expressed anti‐GFP nanobodies is not sufficient to abolish binding in *C. elegans* cells and we describe the rational engineering of nanobody paratopes to overcome this problem by tuning binding affinity of the nanobodies to their target protein. This allows us to optically control binding through incorporation of the photocaged tyrosine variants ONBY and NPY (Figure 1A). We show that the introduction of a photocaged non‐canonical amino acid (ncAA) alone is not sufficient to break nanobody/antigen binding *in vivo*. We perform Computational Alanine Scanning (CAS) to identify surface residues on the nanobodies that contribute to binding and use this information to guide engineering of nanobodies by introducing alanine mutations in place of interaction hot‐spot residues so that binding is abolished in the photocaged form and restored on uncaging. We demonstrate the general applicability of our approach by engineering two anti‐GFP nanobodies and using them to control protein localisation in a living animal, the nematode worm *C. elegans*.

## Results

### Incorporating photocaged tyrosine in *C. elegans*

We set out to photocage nanobodies for use in *C. elegans*, initially focussing on a well‐established anti‐GFP nanobody, the “enhancer” nanobody (eNB),^[39]^ as it is utilised for many *in vivo* tools[^13^, ^14^, ^22^, ^23^, ^24^] and has successfully been expressed and shown to bind GFP in *C. elegans*.^[13]^ It has been shown using *in vitro* cell surface binding assays that replacing eNB residue Y37, which is located in the GFP binding interface, with ortho‐nitrobenzyl tyrosine (ONBY), a photocaged form of tyrosine, can reduce binding affinity 10,000 fold (Figure 1).[^32^, ^40^] In contrast, the alternative photocaged tyrosine nitropiperonyl tyrosine (NPY) (Figure 1A) was previously found to only reduce binding by 420 fold.^[32]^ The nitropiperonyl group on NPY has a red shifted absorption maximum and improved quantum yields compared to the ortho‐nitro group on ONBY. The resulting improved efficiency of uncaging at 365 nm makes NPY preferable for *in vivo* applications.^[41]^ ONBY and NPY have been used to optically control nanobody binding in human cell lines.[^36^, ^37^]

To express photocaged nanobodies *in vivo*, we first established a system for incorporation of photocaged tyrosine in *C. elegans*. Genetic code expansion utilises orthogonal aminoacyl synthetase / tRNA pairs to site specifically direct incorporation of non‐canonical amino acids (ncAA) into target proteins. The most widely applied orthogonal genetic code expansion system is based on the pyrrolysyl synthetase (PylRS) / tRNA(Pyl) from methanogenic archaea, which has been used to expand the genetic code of bacteria, eukaryotic cultured cells, plants, and animals.^[42]^ In *C. elegans*, incorporation of ncAA is possible in all tissues and photocaged amino acids have been used to control protein function in living animals.[^43^, ^44^]

Several caging groups have been used to photocage tyrosine and orthogonal aminoacyl‐tRNA‐synthetase / tRNA pairs have been described in the literature to incorporate them,[^33^, ^41^] albeit thus far no method for the site specific incorporation of photocaged tyrosine has been reported for multicellular organisms. We therefore first established a method for the incorporation of the photocaged tyrosines NPY and ONBY in *C. elegans*. We based our incorporation system on the pyrrolysyl‐tRNA synthetase variant NBYRS, which has the advantage that it recognises, and thus can be used to incorporate, both ONBY and NPY amino acids.^[41]^

We introduced the previously described NBYRS mutations into a *Methanosarcina mazei* (Mm) PylRS gene, optimised for expression in *C. elegans* to make MmNBYRS. We then created NES‐PKIα::MmNBYRS by adding a nuclear‐export sequence (NES) derived from human PKIα to the N‐terminus of MmNBYRS to increase cytoplasmic localisation, a modification which significantly improves incorporation efficiency in eukaryotic cells.[^44^, ^45^] Second, in place of wild‐type tRNA(Pyl)_CUA_ we used the tRNA(C15)_CUA_ variant, which improves incorporation efficiency in mammalian cells and in *C. elegans*.[^44^, ^46^] We showed previously that, in combination, these two improvements can increase incorporation of ncAA in *C. elegans* tissues by more than 50‐fold.^[44]^

**Figure 2:**
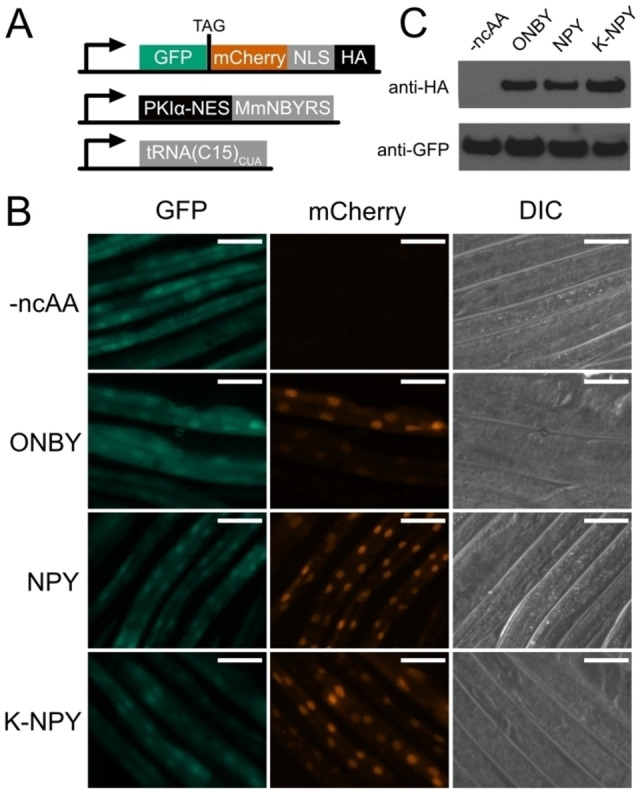
A. Genetic constructs for the fluorescent ncAA incorporation reporter and the machinery for incorporation of photocaged tyrosines. Successful incorporation at the TAG codon will result in the production of full length GFP::mCherry fusion protein. B. Fluorescence imaging of *C. elegans* expressing the fluorescent reporter construct and the ncAA incorporation machinery, in the absence of ncAA and in the presence of 0.1 mM ONBY, NPY, or K‐NPY. Scale bars 50 μm. C. Anti‐GFP and anti‐HA Western blots performed on lysates of the *C. elegans* shown in (B), grown in the absence of ncAA or in the presence of 0.1 mM ONBY, NPY, or K‐NPY.

We generated transgenic strains ubiquitously expressing a fluorescent reporter for incorporation along with the genetic code expansion machinery components NES‐PKIα::MmNBYRS and tRNA(C15)_CUA_. The reporter consists of an N‐terminal GFP separated by a TAG codon from a C‐terminal mCherry fused to the *C. elegans* EGL‐13 nuclear localisation sequence (NLS)^[47]^ followed by an HA tag. The reporter is designed so that prior to incorporation, only GFP is expressed, which is localised throughout the cell, while incorporation of a ncAA at the TAG codon will result in the production of full length GFP::mCherry::NLS protein that is localised to the nucleus (Figure 2A).

Since the reporter construct contains an internal stop codon, it is a target for nonsense mediated decay (NMD). In order to increase the amount of reporter mRNA and ensure easy visualization of incorporation, we generated transgenic strains in the *smg‐6(ok1794)* deletion background, which makes the animal deficient for NMD.

We tested incorporation efficiency by transferring transgenic animals to Nematode Growth Medium (NGM) agar plates containing ncAA (0.1 mM ONBY or 0.1 mM NPY). We observed strong red fluorescence in the nucleus, appearing within 24 hours after introduction to ncAA, indicating successful incorporation (Figure 2B). We further confirmed the identity of the full‐length reporter protein by western blot using antibodies against the C‐terminal HA tag (Figure 2C, Supporting Figure S1). We found that both the ONBY and NPY amino acids were incorporated with comparable efficiency (Figure 2C). Previous reports indicate that supplying ncAA to *C. elegans* as dipeptides may increase uptake of the compounds.^[48]^ We therefore supplied NPY as a K‐NPY dipeptide with lysine attached to the NPY amino group. However, we did not see increased incorporation for K‐NPY as compared to NPY (Figure 2C). Nevertheless, we found that in our buffer conditions NPY was not soluble at concentration above 0.1 mM, while K‐NPY was easily soluble into the mM range. While the dipeptide did not improve amino acid incorporation efficiency, higher ncAA concentrations may help to increase production of photocaged proteins in cases where baseline incorporation efficiency is low. In all following experiments NPY is supplied to the animals in the dipeptide form K‐NPY.

### NPY and ONBY substitution is not sufficient to photocage antigen binding in *C. elegans*

We proceeded to express the anti‐GFP nanobody eNB containing either of the photocaged tyrosines ONBY or NPY in lieu of the native Y37 residue, which is situated at the binding interface.^[32]^ Since both ncAA showed efficient incorporation, we decided to construct transgenic strains for eNB expression in the wild type N2 strain, which has functional NMD.

**Figure 3:**
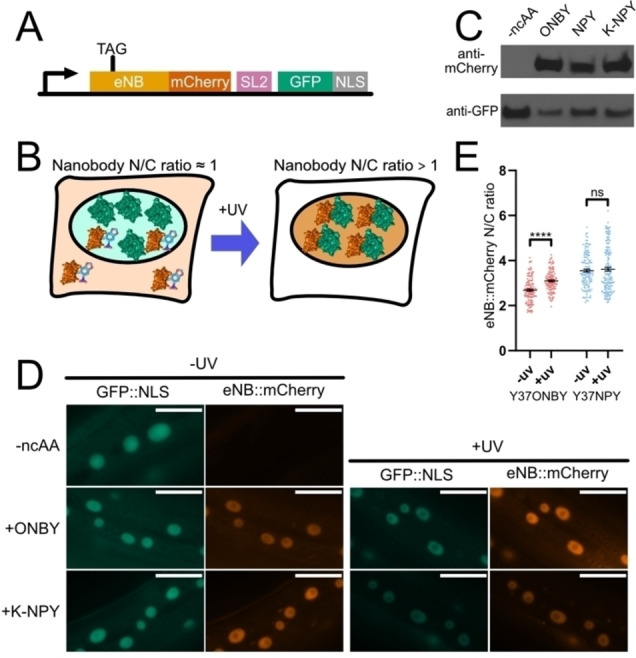
A. Genetic construct for the eNB/GFP interaction reporter. eNB::mCherry is produced upon incorporation of ncAA at the TAG codon. GFP fused to a nuclear localisation sequence is expressed in an artificial operon behind the same promoter independently of ncAA incorporation. B. Schematic of the *in vivo* photoinducible interaction assay. Photocaged nanobodies fused to mCherry are co‐expressed with nuclear GFP. If incorporation of a photocaged amino acid into the GFP‐binding region of the nanobody is sufficient to abolish GFP binding, the mCherry nuclear/cytoplasmic ratio (N/C) will be near 1, as measured by mCherry fluorescence. Following uncaging with 365 nm light (+UV), the nanobody will regain its ability to bind GFP and relocate to the nucleus, leading to an increase in the N/C ratio. C. Anti‐GFP and anti‐mCherry Western blots performed on lysates from *C. elegans* expressing the interaction reporter constructs described in 3A and grown in the absence of ncAA or in the presence of 0.1 mM ONBY, NPY, or K‐NPY. D. Images of cells in animals of the *in vivo* assays of the interaction between GFP and eNB^wt^, eNB^Y37ONBY^, or eNB^Y37NPY^, before and after 365 nm illumination. Scale bars 20 μm. E. Quantification of mCherry nuclear/cytoplasmic ratio for eNB with mutations Y37ONBY or Y37NPY. Data are presented as measurements of individual cells and mean ± SEM. Measurements were taken from 7–10 animals per condition. Difference between means of ‐UV and +UV was assessed using a two‐tailed unpaired Welch's t‐test. ns p>0.05; **** p<0.0001.

To assay eNB to GFP binding *in vivo*, under physiological conditions, we employed a subcellular localisation assay as a visual read‐out. We expressed a eNB::mCherry fusion protein with mCherry attached to the eNB C‐terminus. We furthermore co‐expressed GFP fused to the *C. elegans* EGL‐13 nuclear localisation signal (NLS)^[47]^ (Figure 3A), so that binding of eNB::mCherry to GFP would result in the nuclear localisation of the red mCherry signal. We reasoned that GFP binding strength of different eNB variants (with and without photocaged residues) could be compared by measuring the nuclear to cytoplasmic ratio (N/C) of the eNB::mCherry fusion. Strong binders would be mostly nuclear, whereas non‐binders would be free to diffuse throughout the cell (Figure 3B). The eNB::mCherry fusion has a molecular weight of around 40 kDa, a size which easily allows free diffusion across the nuclear membrane.^[49]^ Additionally, the C‐terminal mCherry would act as a reporter for successful ncAA incorporation. We confirmed that the wild‐type eNB::mCherry (i. e. not containing any photocaged residues) indeed localises to the nucleus *in vivo* and therefore binds to GFP::NLS. Conversely, co‐expression of eNB::mCherry with NLS::mTagBFP2, a blue fluorescent protein which is not related to GFP, does not lead to nuclear localisation of eNB::mCherry. Likewise, mCherry alone, without a fused eNB, does not localise to the nucleus when co‐expressed with GFP::NLS (Supporting Figure S2).

To direct incorporation of ONBY and NPY, we introduced a TAG stop codon replacing the codon for Y37 into eNB. We co‐expressed eNB(TAG)::mCherry together with GFP::NLS and the components of the photocaged tyrosine incorporation machinery, consisting of NES‐PKIα::MmNBYRS and tRNA(C15)_CUA_.

The transgenic *C. elegans* strains we generated showed a strong GFP signal that was exclusively nuclear. As expected, we saw no mCherry signal in the absence of ncAA. When we transferred the animals to plates supplemented with 0.1 mM ONBY, NPY, or K‐NPY we saw clearly visible red fluorescence appearing within 24 h, indicating production of the full length eNB^Y37ONBY^::mCherry or eNB^Y37NPY^::mCherry fusion proteins, we confirmed the production of full length protein by western blot for mCherry (Figure 3C, Supporting Figure S1). As with the GFP::mCherry incorporation reporter we saw no difference in incorporation efficiency between the ncAAs, even when supplying NPY as the dipeptide K‐NPY.

To our surprise, we found that both photocaged nanobodies eNB^Y37ONBY^ and eNB^Y37NPY^ co‐localised with GFP::NLS in the nucleus (Figure 3D). This observation was unexpected since previously published *in vitro* binding assays, using photocaged eNB purified from *E. coli*, showed that Y37 substitution for ONBY reduces binding affinity 10,000 fold and blocks binding *in vitro*, and NPY reduces binding affinity 420 fold.^[32]^ Furthermore, photo‐caging of eNB using ONBY or NPY resulted in blocking of target binding when nanobody and target were co‐expressed in cultured HeLa cells.[^36^, ^37^]

While ONBY was not sufficient to abolish binding in our intracellular assay, we found that uncaging by illuminating animals using a 365 nm LED (640 seconds illumination, 10 mW/cm^2^) led to a clear increase in the N/C ratio for eNB^Y37ONBY^ from 2.69 (±0.05) to 3.10±(0.04) (Figure 3E, Supporting Figure S3). This contrasts with eNB^Y37NPY^, where there was no significant change in N/C, going from 3.55 (±0.07) before illumination to 3.61 (±0.08) after uncaging. Our *in vivo* observations therefore support the previous *in vitro* observation that ONBY is more disruptive to the interaction than NPY. Neither the disruption by ONBY, or by NPY, is however sufficient to block binding in our intracellular assay.

### Molecular dynamics simulations and in silico alanine scan for tuning of the eNB/GFP interaction

Since the sole introduction of a caging group was insufficient to fully disrupt binding in our assay in *C. elegans*, we required a means to further reduce binding. Several factors can influence PPI and complex formation: the concentration of the binding partners, their binding affinity, and the buffer or cellularenvironments. While buffer conditions can easily be controlled *in vitro*, this possibility does not exist *in vivo*. Likewise, controlling the concentration of the binding partners, which also can be easily achieved *in vitro*, is far more challenging *in vivo* where protein levels may be influenced by a combination of factors such as cell to cell stochastic variability^[50]^, promoter strength and cellular degradation pathways, and, especially in multicellular organisms, are subject to dynamic regulation by developmental stage and environmental conditions.

We therefore reasoned that the most straightforward approach would be further engineering of the nanobody/antigen binding interface to yield photoinducible nanobodies which function robustly in complex intracellular environments. Our aim was to reduce the binding strength so that the introduction of a photocaged amino acid would abolish binding, yet upon uncaging the nanobody would retain sufficient affinity to bind its target.

**Figure 4:**
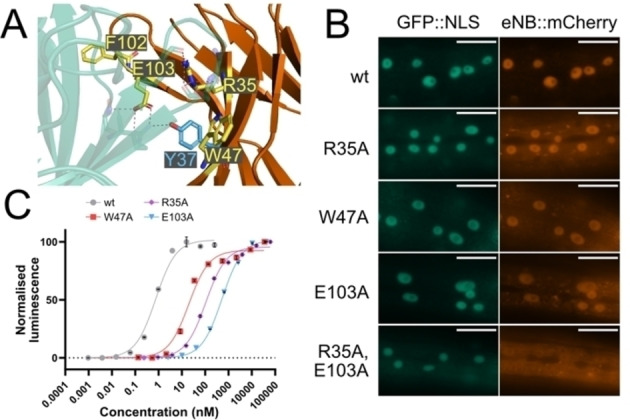
A. eNB/GFP interface (PDB: 3 K1 K), with eNB residues that are predicted by BAlaS to have the greatest contribution to GFP binding highlighted in yellow. B. Images of cells in *C. elegans* expressing GFP::NLS and eNB::mCherry with different alanine mutations. Scale bar 20 μm. C. ELISA assays of eNB mutants against GST::sfGFP. Data are presented as means of three repeats per eNB concentration ± SEM (where SEM is large enough to display). The data were fit to a sigmoidal nonlinear curve using the GraphPad Prism 9.1 pre‐set analysis option “One site ‐ specific binding”

Since nanobodies bind their antigen via multiple interactions, we hypothesised that removing interactions between eNB and GFP in addition to Y37 photocaging would result in such an eNB variant suitable for intracellular use (Figure 4A). To develop a universally applicable approach to identify suitable candidate residues for this purpose, we turned to computational alanine scanning (CAS).

Computational Alanine Scanning (CAS) is a generally applicable technique for identifying hot‐spot residues or sets of residues that contribute to binding interactions. CAS programs are *in silico* alternatives to classic alanine scanning mutagenesis experiments, which can be prohibitively time‐consuming and costly. BUDE Alanine Scan (BAlaS) is an open‐source, state‐of‐the‐art CAS program freely available for use via an in‐browser application.[^51^, ^52^] BAlaS calculates the binding free energy (ΔG) of a given protein‐protein interaction (PPI), for the native protein sequence and for when each residue is independently mutated to alanine. The software accepts protein crystal structures or multi‐model files such as NMR ensembles and molecular‐dynamics trajectories. We used BAlaS to perform *in silico* alanine scanning on eNB in complex with GFP to generate a shortlist of alanine mutations predicted to decrease binding strength. We hypothesised that introducing such alanine mutations into eNB would reduce binding affinity and this, in combination with photocaging of Y37, would make the eNB/GFP interaction photoinducible.

BAlaS has been demonstrated to generate more accurate predictions of hot‐spot residues when multiple conformations of the protein are provided.^[52]^ To generate input data for BAlaS, we first ran twenty independent 20 ns molecular‐dynamics (MD) simulations for each nanobody variant: eNB^wt^, eNB^Y37ONBY^, and eNB^Y37NPY^. Electronic parameters for the ONBY and NPY residues were derived using the Generalised Amber Force Field,^[53]^ except for the nitro group, where values from quantum mechanical calculations were used.^[54]^ Simulations were run using the OpenMM software.^[55]^ The simulations rapidly reach equilibrium, after which point the temperature and energy remains constant (Supporting Figure S4). The final frames from each of the twenty simulations were used as inputs for BAlaS, representing a sample of twenty representative poses the complex can adopt. The output ΔG value for each variant is the mean binding free energy of these twenty poses.

BAlaS predicted ΔGs of the three eNB variants eNB^wt^, eNB^Y37ONBY^, and eNB^Y37NPY^ that are in line with the experimental results of ourselves and others^[32]^ that ONBY is more disruptive to the interaction than NPY (Supporting Figure S6), supporting the robustness of our *in silico* method.

Close examination of the MD trajectories offers explanations for why, surprisingly, ONBY is more disruptive than the bulkier NPY. In the crystal structure and MD trajectories of the eNB^wt^/GFP interaction, Y37 of eNB forms a hydrogen bond with R168 of GFP (Supporting Figure S7). This makes Y37 an obvious candidate for photocaging, and in every eNB^Y37ONBY^ and eNB^Y37NPY^ trajectory this hydrogen bond is abolished. However, in 38 out of the 40 photocaged eNB trajectories, the photocaging groups form a stable cation‐pi interaction with the positively charged guanidinium group of R168 on GFP, the two groups aligning in parallel to one another and packing tightly (Supporting Figure S7). The stronger GFP binding of eNB^Y37NPY^ compared with eNB^Y37ONBY^ could be due to the caging groups’ relative abilities to interact with GFP's R168.

To identify further eNB residues important for GFP binding, which could be combined with photocaged Y37 to make the binding photoinducible, we performed a computational alanine scan on eNB^wt^ using BAlaS. The scan identified 28 eNB residues that are predicted to lower binding strength when mutated to alanine. The effects on the binding energy ranged from +0.01 kJ/mol to +9.62 kJ/mol (Supporting Figure S8). The most disruptive alanine mutations predicted were R35A, Y37A, W47A, F102A and E103A, indicating that the corresponding residues are important for GFP binding. All residues are situated within the eNB/GFP interface, except F102, which is at the periphery of the interface (Figure 4A). To test the effects of these mutations in worm cells, we generated *C. elegans* strains co‐expressing GFP::NLS and eNB::mCherry fusions where eNB is mutated with one of R35A, W47A, or E103A, or doubly mutated with R35A and E103A. eNB^R35A^, eNB^W47A^, and eNB^E103A^ all displayed GFP binding, whereas eNB^R35A, E103A^ displayed no binding (Figure 4B). We performed ELISA assays against sfGFP to compare the binding strength of the single alanine mutants to that of wild‐type eNB (Figure 4C). For eNB^wt^ *K_d_=*0.76±0.12 nM (R^2^=0.9895), for eNB^R35A^ *K_d_=*106.10±11.56 nM (R^2^=0.9956), for eNB^W47A^ *K_d_=*19.82±3.75 nM (R^2^=0.9830), and for eNB^E103A^ *K_d_=*552.10±27.10 nM (R^2^=0.9991). The mutations significantly decreased eNB‐GFP binding *in vitro* by between 26‐fold for W47A and 726‐fold for E103A, but this decrease was not sufficient to abolish binding in our intracellular assay.

### Use of engineered photocaged eNB variants in *C. elegans*

We proceeded to test whether the disruptive mutations in combination with photocaging Y37 could indeed be used to engineer photocaged nanobodies. For this, we combined the single mutations R35A, W47A, or E103A, with a TAG codon at position Y37 to direct incorporation of photocaged tyrosines. We also generated a triple mutant combining the R35A and E103A mutations with the TAG mutation at Y37. We then constructed transgenic *C. elegans* strains expressing the MmNBYRS / tRNA (C15)_CUA_ ncAA incorporation machinery together with the eNB::mCherry mutants and GFP::NLS.

We decided to test eNB variants using the NPY caged tyrosine, since it is more amenable to uncaging at 365 nm than ONBY,^[41]^ and would therefore be preferable for *in vivo* usage. To incorporate NPY, we transferred animals to plates supplemented with 0.1 mM K‐NPY. 24 h after transfer, we observed the appearance of red fluorescence indicative of NPY incorporation and production of full length eNB::mCherry protein.

**Figure 5:**
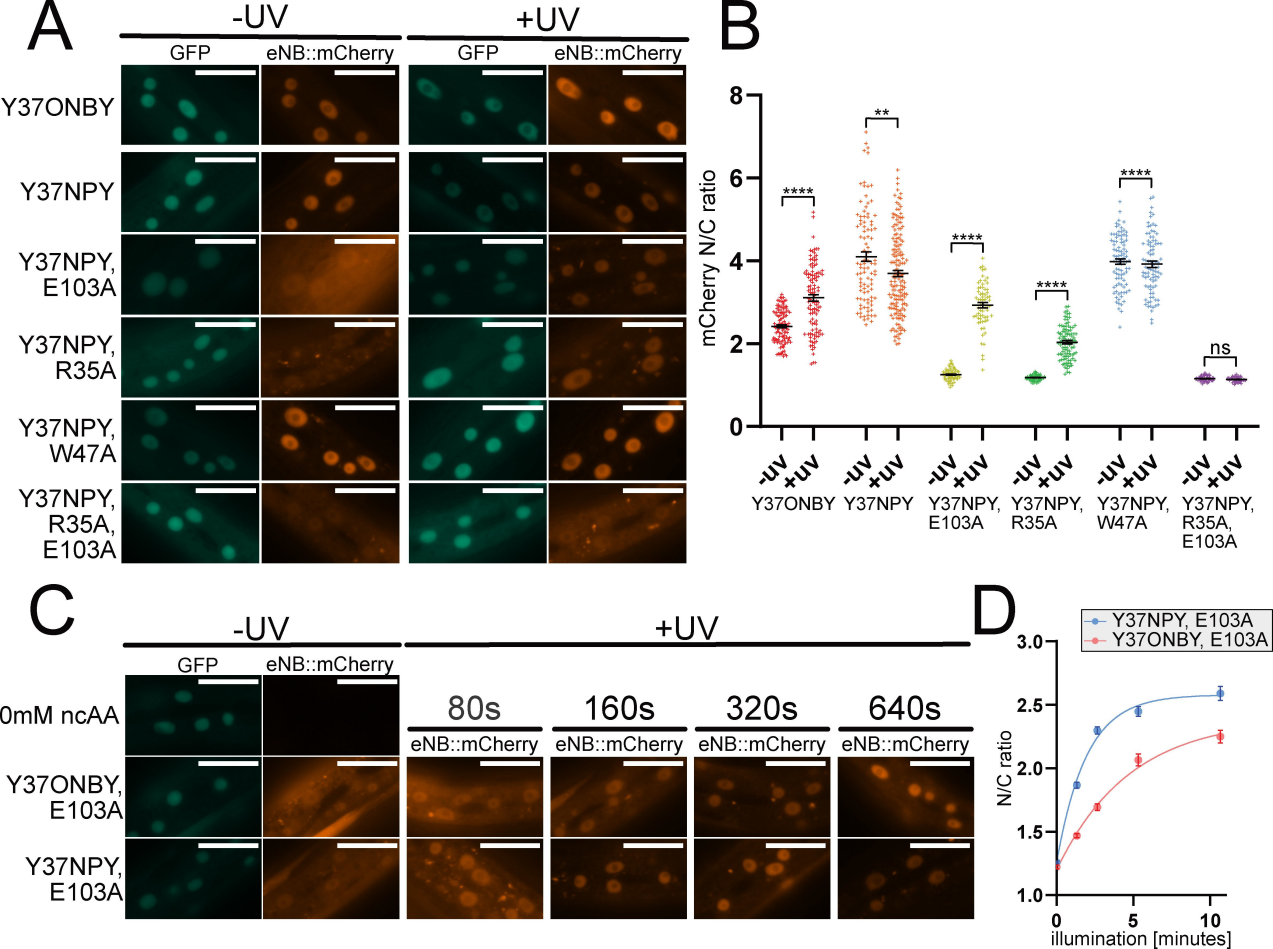
A. Images of cells in *C. elegans* expressing GFP::NLS with different photocaged eNB variants, before and after 365 nm illumination. Scale bar 20 μm. B. Quantification of mCherry nuclear/cytoplasmic ratio of photocaged eNB variants before and after 365 nm illumination. Data are presented as individual cell measurements and mean±SEM. Measurements were taken from 7–10 animals per condition. Difference between the means for ‐UV and +UV of each nanobody was assessed using a two‐tailed unpaired Welch's t‐test. ns p>0.05; ** p<0.01; *** p<0.0001. C. Images of cells in animals expressing eNB^Y37ONBY, E103A^ and eNB^Y37NPY, E103A^, subjected to a range of 365 nm illumination times. Scale bar 20 μm. D. Quantification of mCherry nuclear/cytoplasmic ratio of eNB^Y37ONBY, E103A^ and eNB^Y37NPY, E103A^ subjected to a range of 365 nm illumination times. Data are presented as mean±SEM. Measurements were taken from 7–10 animals per condition. Difference between ONBY and NPY means for each illumination time was assessed using a two‐tailed unpaired Welch's t‐test. ONBY vs NPY: 0 s p=0.2151, all other time points p<0.0001; ONBY 640s vs NPY 160s p=0.4110.

In stark contrast to animals expressing eNB^Y37ONBY^ and eNB^Y37NPY^, where the red eNB::mCherry fluorescence was predominantly localised to the nucleus, we found that for the variants eNB^Y37NPY, R35A^, eNB^Y37NPY, E103A^, and eNB^Y37NPY, R35A, E103A^ the red fluorescence was distributed throughout the cell, indicating that the introduction of additional mutations were sufficient to abolish eNB to GFP binding. Interestingly, the eNB^Y37NPY, W47A^ variant retained strong GFP binding as evidenced by its clear nuclear localisation (Figure 5A).

We proceeded to test whether removal of the caging group would re‐establish eNB/GFP binding by quantifying the eNB::mCherry N/C ratio before and after uncaging. We illuminated animals using a 365 nm LED and compared these with unilluminated animals. Upon illumination we observed a striking shift in localisation to the nucleus for the eNB^Y37NPY, R35A^ and eNB^Y37NPY, E103A^ variants (Figure 5A, 5B). Illumination induced an increase in the N/C ratio of eNB^Y37NPY, R35A^ from 1.18 (±0.01) to 2.04±(0.04), and of eNB^Y37NPY, E103A^ from 1.26 (±0.02) to 2.59 (±0.07) (Figure 5B, Supporting Figure S9). eNB^Y37NPY, W47A^ remained nuclear with no significant change, going from 3.98 (±0.07) to 3.92 (±0.07). eNB^Y37NPY, R35A, E103A^ remained cytoplasmic following uncaging, its N/C ratio going from 1.16 (±0.01) to 1.14 (±0.01), indicating that introduction of two alanine mutations abolished the ability of the eNB variant to bind GFP even after removal of the photo‐caging group.

We then compared ONBY and NPY to test the effect of different photocaging groups on the effectiveness of our approach of tuning the interaction strength. For this, we grew animals expressing the eNB^Y37TAG, E103A^ variant on NGM agar plates supplemented with either 0.1 mM ONBY or 0.1 mM K‐NPY. In both cases we observed the appearance of red fluorescence that was distributed throughout the cell, indicating the presence of eNB^Y37ONBY, E103A^ or eNB^Y37NPY, E103A^ (Figure 5C). Interestingly, there was no significant difference between N/C ratios of eNB^Y37ONBY, E103A^ and eNB^Y37NPY, E103A^ before uncaging, with values of 1.23 (±0.01) and 1.26 (±0.01) respectively (Figure 5D, Supporting Figure S10). Upon uncaging using a 365 nm LED, red fluorescence accumulated in the nucleus for both variants. However, we saw a pronounced difference in the uncaging kinetics, with ONBY requiring significantly longer illumination times than NPY. eNB^Y37ONBY, E103A^ required 640s illumination to reach the N/C ratio of 2.26 (±0.05), while eNB^Y37NPY, E103A^ reached an N/C ratio of 2.30 (±0.03) with a quarter of the illumination time. This reflects the fact that NPY has a red‐shifted absorption maximum compared to ONBY and is therefore more amenable to uncaging using 365 nm.^[41]^

### Development of photocaged mNB

**Figure 6:**
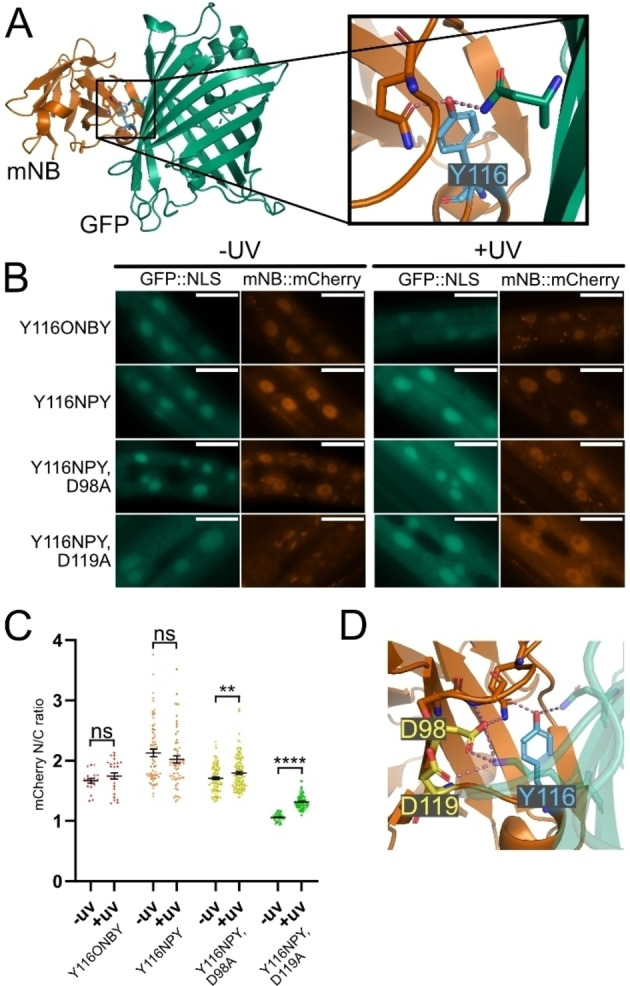
A. Crystal structure of mNB/GFP interaction (PDB: 3G9 A). Highlighted in blue is Y116 of mNB, which forms a polar interaction with R168 of GFP. B. Images of cells in *C. elegans* expressing GFP::NLS with different photocaged mNB variants, before and after 365 nm illumination. Scale bar 20 μm. C. Quantification of mCherry nuclear/cytoplasmic ratio of photocaged mNB variants before and after 365 nm illumination. Data are presented as individual cell measurements and mean±SEM. Measurements were taken from 7–10 animals per condition. Difference between the means for ‐UV and +UV of each nanobody was assessed using a two‐tailed unpaired Welch's t‐test. ns p>0.05; ** p<0.01; **** p<0.0001. D. mNB/GFP interface (PDB: 3G9 A) with two mNB residues that are predicted by BAlaS to have significant effect on interaction strength when mutated to alanine highlighted in yellow.

After successfully modifying eNB, we proceeded to test whether our approach was generally applicable by engineering a second nanobody, the “minimiser” GFP‐binding nanobody (mNB).^[39]^ We chose Y116 on mNB as a candidate residue for substitution with a photocaged tyrosine to achieve photoinducible GFP binding. Y116 is in the centre of the binding interface and forms hydrogen bonds with GFP N164 and mNB Q102 (Figure 6A). The introduction of ONBY in place of this residue has previously been shown by Jedlitzke et al. to moderately affect binding *in vitro*.^[32]^

We specified incorporation of photocaged amino acids by introducing a TAG codon at position Y116 and generated *C. elegans* strains co‐expressing the mNB^Y116TAG^ variant together with the photocaged tyrosine incorporation machinery. To quantify GFP binding of the photocaged mNB we again used our *in vivo* assay where photocaged mNB::mCherry fusion is co‐expressed with GFP::NLS in live animals. Surprisingly, we found that the incorporation efficiency into mNB was lower than for eNB. We therefore used the NMD deficient *smg‐6(ok1794)* deletion background to assay mNB variants. Furthermore, we took advantage of higher solubility of the K‐NPY dipeptide and grew animals for NPY incorporation on 2 mM K‐NPY plates. Upon imaging worms grown on 0.1 mM ONBY and 2 mM K‐NPY, we found that both mNB^Y116ONBY^ and mNB^Y116NPY^ localised to the nucleus prior to uncaging (Figure 6B). In neither case was a significant change in localisation observed following uncaging (Figure 6C). This result indicates that, as we previously observed with eNB, introducing a photocaged tyrosine to the interface was not sufficient to break the nanobody/antigen interaction *in vivo*.

We proceeded to run MD simulations followed by BAlaS scans and found several alanine mutations with effects on GFP binding ranging from +0.02 kJ/mol to +17.90 kJ/mol (Supporting Figure S11). We selected two mutations, D98 A and D119 A (Figure 6D), and introduced them separately into mNB alongside the Y116 amber mutation. We generated transgenic *C. elegans* strains expressing these mNB mutant variants together with the incorporation machinery, and GFP::NLS. We then grew transgenic animals on plates supplemented with 2 mM K‐NPY to express mNB^Y116NPY, D98A^ or mNB^Y116NPY, D119A^, respectively. We found that mNB^Y116NPY, D98A^ was nuclear before uncaging, with an N/C of 1.71 (±0.22), indicating that the addition of a D98 A alanine mutation was not sufficient to break the interaction (Figure 6B, 6 C). In contrast, we found that the mNB^Y116NPY, D119A^ nanobody variant was distributed uniformly throughout the cell and translocated to the nucleus following uncaging, with N/C going from 1.06 (±0.06) to 1.31 (±0.11). The D119A mutation in addition to Y116NPY was therefore sufficient to break mNB/GFP binding, while uncaged mNB^D119A^ retained sufficient affinity to bind to GFP.

## Discussion

Nanobodies are small, highly specific protein binders, which have been exploited to develop a vast range of *in vivo* tools for investigating biological processes. Several avenues have been explored to date to develop methods for making nanobody antigen binding inducible. The ability to control binding with light would provide the greatest spatiotemporal control of nanobody/antigen interaction, but progress to date has been unsatisfactory, with problems such as slow kinetics, background activity, and a dependence on blue light. The use of photocaged amino acids to control nanobody/antigen binding may help to overcome these limitations as uncaging is rapid and the photocaging groups are stable in blue light, making their application compatible with commonly used imaging and optogenetic approaches.

In principle, the introduction of photocaged residues into binding interfaces can be used to disrupt the formation of PPI, while removal of the photocaging group will restore the native protein and therefore its native binding ability. A difficulty in using photocaged amino acids for this purpose is that replacement of a native residue with its caged counterpart alone may not be sufficient to entirely break the PPI, especially in intracellular environments, thus resulting in considerable residual binding. Here we provide a solution to this problem by combining rational interface engineering, guided by computational alanine scanning and molecular dynamics simulations, with the site‐specific incorporation of photocaged ncAA to design improved variants of two anti‐GFP nanobodies for use in cellular environments. The introduction of the photo‐caged tyrosines ONBY or NPY into the improved variants abolishes binding to GFP in the intracellular environment within a living animal, and removal of the caging group upon illumination restores binding. Our approach will be applicable to other nanobodies and will also allow the control of the binding interaction through tuning and photo‐caging of the target protein.

We establish the use of the photocaged tyrosines ONBY and NPY in *C. elegans* using an enhanced genetic code expansion machinery.[^33^, ^41^, ^44^, ^45^, ^46^] This represents the first instance of caged tyrosine variants being successfully incorporated in a multicellular organism. We achieve efficient incorporation even in a wild‐type genetic background and therefore in the presence of a functional nonsense mediated decay machinery.

The photocaged tyrosine ONBY has previously been used to photocage nanobody/antigen binding *in vitro*, when exogenously delivered to cells,[^32^, ^34^] and when expressed in HeLa cells.[^36^, ^37^]

We have found that the introduction of an ONBY residue into the GFP binding interface of eNB is not sufficient to break binding when photocaged nanobody and antigen are expressed together in *C. elegans*. Our results show that developing a photo‐controllable nanobody for use inside living cells may require additional engineering steps beyond the simple introduction of a photocaged amino acid residue. *In vitro* conditions where proteins are in a controlled, dilute buffer often do not fully replicate conditions encountered in the crowded interior of cells where the environment is composed of a large variety of tightly packed macromolecules.^[56]^ Factors such as nanobody concentration, the intracellular “incubation” time (which begins from the time of expression and may therefore be longer than that of *in vitro* experiments), and the effects of macromolecular crowding may all contribute to the failure of the photocaged tyrosines to break binding in an intracellular environment.

We have encountered this phenomenon for both of the anti‐GFP nanobodies we tested, suggesting that the introduction a photocaged amino acid alone may not be sufficient for the development of photocaged nanobodies for all applications. Our data suggest that even nanobodies with reduced binding affinities can be competent binders in intracellular environments. Therefore, the exceptionally strong antigen affinity of nanobodies may not a required or even desirable trait when engineering photo‐activatable nanobodies for intracellular *in vivo* use as it may result in substantial photocaged nanobody/antigen interaction pre‐activation. Indeed, molecular‐dynamics simulations of the wild‐type and photocaged nanobodies presented here, suggest that the photocaging groups themselves can add stabilising interactions with the antigen that contribute to the binding we observed. This highlights the complex nature of these interactions and demonstrates the value of a rigorous computational approach to engineering interfaces such as these. Furthermore, our data show the importance of *in vivo* validation, in the relevant model system, for *in silico* PPI predictions and *in vitro* PPI measurements.

Our method represents a novel application of Computational Alanine Scanning (CAS) and a rare example of the results being experimentally validated in a system as complex as a living animal. Our *in vivo* localisation‐based binding assay provides a simple, effective method to validate computationally predicted interaction hot‐spots in physiologically relevant environments. We believe our work demonstrates that *in vivo* validation methods should be considered when designing hot‐spot targeted drugs and antibodies.

Of the nanobody mutants tested, three out of six resulted in functional photoinducible nanobodies. Interestingly, one of the mutations that failed to break binding, W47 A in eNB, was predicted by BAlaS to have the largest contribution to the free‐energy of binding. This might indicate a bias in the scoring function used in BAlaS, which results in over weighting of polar residues vs nonpolar residues. BAlaS was found to produce more accurate results when the contributions of polar residues (DERKH) are down‐weighted.^[52]^ Our observations indicate that this correction could be too severe. As noted in the Results, BAlaS suggested Y37 as an important residue, which also indicates that CAS could be used to identify candidate residues to photocage. In future, we plan to further develop this pipeline to fully automate the process of selecting the identity and location of the photocaged residue, as well as possible secondary mutations to tune the interaction strength, to make the process of generating light‐inducible interactions more accessible to the broader scientific community.

Importantly, by tuning the binding energy through mutations informed by our *in silico* alanine scanning approach, we were able to photocage nanobody/GFP binding using the photocaged tyrosine NPY. NPY is more amenable to *in vivo* applications than ONBY due to its superior uncaging properties, with a red shifted absorption maximum of 365 nm compared to 254 nm for ONBY. Using our approach we were able to generate photo‐controllable variants of the anti‐GFP enhancer nanobody (eNB), which is used as the basis of a wide range of *in vivo* tools.[^2^, ^13^, ^14^, ^22^, ^23^, ^24^] These variants may thus immediately add the ability to use light for the precise spatiotemporal control of these tools, with the photocaged tyrosine that is most amenable to *in vivo* systems, NPY.

## Conclusion

In conclusion, we have developed a generally applicable method for the rational engineering of photo‐activatable nanobodies and we apply it to intracellularly expressed nanobodies in a living animal. We have shown the feasibility of tuning binding energies through the targeted introduction of alanine mutations into the binding interface as directed by CAS. A general method for the engineering of photocaged nanobodies will allow the addition of precise spatiotemporal control to the large and growing number of existing nanobody based tools, greatly enhancing their power to drive discovery in the large number of model systems in which they are employed.

## Conflict of interest

The authors declare no conflict of interest.

1

## Supporting information

As a service to our authors and readers, this journal provides supporting information supplied by the authors. Such materials are peer reviewed and may be re‐organized for online delivery, but are not copy‐edited or typeset. Technical support issues arising from supporting information (other than missing files) should be addressed to the authors.

Supporting Information

## Data Availability

The data that support the findings of this study are available in the supplementary material of this article.
