# Supplementary material for: Generation of Photocaged Nanobodies for Intracellular Applications in an Animal Using Genetic Code Expansion and Computationally Guided Protein Engineering
Source: Chembiochem. 2022 Jul 7;23(16):e202200321. doi: 10.1002/cbic.202200321 (PMC9542635; doi:10.1002/cbic.202200321)
Supplement: Supplementary file 1 — Supporting Information [file CBIC-23-0-s001.pdf]

ChemBioChem

Supporting Information

Generation of Photocaged Nanobodies for Intracellular Applications in an Animal Using Genetic Code Expansion and Computationally Guided Protein Engineering**

Jack M. O'Shea, Angeliki Goutou, Jack Brydon, Cyrus R. Sethna, Christopher W. Wood, and Sebastian Greiss*

Methods 2

C. elegans maintenance 2

Plasmid generation 2

C. elegans feeding of photocaged amino acids 2

Uncaging of photocaged nanobodies 3

C. elegans lysis and Western blotting 3

Imaging of *C. elegans* 3

Measurement of mCherry nuclear:cytoplasmic ratio 3

Statistical analysis of mCherry nuclear:cytoplasmic ratio 4

Parameterisation of photocaged amino acids for molecular dynamics 4

Molecular dynamics of nanobody/GFP complexes 4

Alanine scanning of nanobody/GFP complexes 4

Cloning plasmids for *E. coli* periplasmic protein expression 4

Expression and purification of GST::GFP and GST 4

Expression and purification of nanobody mutants 5

ELISAs of nanobody/GFP interactions 6

Supplementary Tables **7**

Transgenic *C. elegans* strains 7

Expression plasmids 7

Destination vectors 8

pENTR P4-P1r vectors 8

pENTR 221 vectors 8

pENTR P2r-P3 vectors 10

G-blocks 11

Bacterial expression plasmids 12

Supplementary Figures **14**

S1 – Western blots of photocaged amino acid incorporation reporters 14

S2 – Images of eNB/GFP binding control strains 15

S3 – Repeats of $eNB^{Y37ONBY}$ and eNB^{Y37NPY} localisation assays 16

S4 – Schematic of nanobody design plan for photoinducibility 17

S5 – Plots of molecular dynamics simulation conditions 18

S6 – BUDE Alanine Scan calculated ΔG values for eNB^{wt}, $eNB^{Y37ONBY}$ and eNB^{Y37NPY} binding to GFP 24

S7 – Representative images of ONBY and NPY alignment during simulations 25

S8 – BUDE Alanine Scan calculated ΔΔG values for eNB alanine mutations 26

S9 – Repeats of alanine-mutant photocaged eNB localisation assays 27

S10 – Repeats of $eNB^{Y37NPY, E103A}$ and $eNB^{Y37ONBY, E103A}$ dose response assays 28

S11 – BUDE Alanine Scan calculated ΔΔG values for mNB alanine mutations 29

References **30**

Methods

C. elegans maintenance

C. elegans strains were maintained under standard conditions unless otherwise stated.[1,2]

Plasmid generation

All plasmids used for transgenesis of *C. elegans* are described in Supplementary Table 1. Plasmids for expression in *C. elegans* were generated using 3 Fragment Multisite Gateway Cloning (Thermo Fisher Scientific).
Details on cloning are listed in Supplementary Table 2. All primers and synthetic genes (gBlocks) were synthesised by IDT. All genes were optimised for expression in *C. elegans* using the online *C. elegans* codon adaptor.[3] PCRs were carried out using Q5 2x Hot-Start Master Mix (New England Biolabs). PCR and digestion products were recovered from agarose gels following electrophoresis using the Zymogen Gel Recovery Kit (Zymo Research). DNA assembly was performed using NEBuilder 2x Master Mix (New England Biolabs). Standard ligation of restriction enzyme digest products was performed using the Roche Rapid Ligation Kit (Merck Life Sciences Life Sciences). All reagents were used in accordance with the manufacturer's specifications.

Entry and expression plasmids were grown in NEB-5α cells (New England Biolabs). Destination plasmids were grown in One Shot *ccdB* survival cells (Thermo Fisher Scientific). Transformations were performed in accordance with the manufacturer's specifications. Transformed cells were grown overnight on TB agar containing the appropriate selection antibiotics. Plasmids were recovered from bacteria using the QIAprep Spin Miniprep kit (Qiagen).

C. elegans transgenesis

Transgenic *C. elegans* strains were generated by biolistic bombardment into either the N2 or *smg-6(ok1794)* genetic backgrounds, with Hygromycin B resistance used as the selection marker as previously described.[4] Spermidine (Merck Life Sciences Life Sciences) was used to precipitate DNA onto gold particles of 0.3-3µm diameter (ChemPur). 900psi rupture discs (Bio-Rad) and macro carrier discs (Inbio Gold) were used in a PDS1000/He Biolistic Particle Delivery System (Biorad). Transgenic strains were maintained on NGM agar supplemented with 0.3mg/mL Hygromycin B (Formedium). All strains used in this study are described in Supplementary Table 3.

C. elegans feeding of photocaged amino acids

The amino acid NPY and the dipeptide K-NPY were custom synthesised (NewChem Technologies), and ONBY was purchased (Fluorochem). K-NPY was dissolved in water and added to molten NGM agar to make the desired concentration. NPY and ONBY were dissolved in 5M NaOH before addition to molten NGM agar, followed by neutralisation by HCl.

Before feeding with ncAAs, worms were grown on NGM agar plates seeded with a lawn of *E. coli* OP50 bacteria. Animals were grown until the food was depleted and the population contained a large number of age synchronised L1 larvae. Synchronised populations of L1 animals were washed off starved plates using M9 buffer and transferred to ncAA-NGM agar plates. 40uL of dissolved freeze-dried OP50 (LabTIE) was added to the plates as food. Animals were grown on ncAA plates at 20°C for 24-48 hours before uncaging followed by imaging or western blotting.

Uncaging of photocaged nanobodies

Animals were uncaged under a 365nm LED with the output set to 30% ($10mW/cm^2$). Animals were transferred from ncAA-NGM agar plates to NGM-only plates before uncaging. Animals were imaged immediately after uncaging.

***C. elegans* lysis and Western blotting**

Synchronised *C. elegans* populations were grown on ncAA-NGM agar plates at 20°C for 24-48 hours, then washed off plates using M9 buffer (supplemented with 0.001% Triton-X100 to prevent animals from sticking to pipette tips). Worms were settled by gravity, the supernatant was removed, and worms were resuspended in lysis buffer at a 1:2 volume ratio of worms to lysis buffer. The lysis buffer consisted of a 4:1 mix of 4X Bolt LDS Sample Buffer (Life Technologies) and NuPAGE Sample Reducing Agent (Thermo Fisher Scientific) respectively. Lysis was performed by a freeze/thaw cycle of overnight freezing at -80°C followed by 15 min incubation at 95°C while shaking.

Samples were run on precast Bolt 4 to 12% gels (Thermo Fisher Scientific) for 18 min at 200V. Proteins were transferred from the gel onto a nitrocellulose membrane using an iBlot2 device (Thermo Fisher Scientific).

After transfer, the membrane was blocked with 5% milk powder in PBST (1xPBS supplemented with 0.1% Tween-20) for 1h at room temperature. Incubation with primary antibodies was carried out in PBST + 5% milk powder at 4C overnight. Blots were washed 4 x 5 minutes with PBST + 5% milk powder before incubation with secondary antibody for 1h at room temperature.

The primary antibodies used were mouse anti-GFP (clones 7.1 and 13.1) (Roche) at a dilution 1:5000 for SGR57 and SGR58, rat anti-HA clone 3F10 (Roche) at a dilution of 1:1000 for SGR57, and mouse anti-mCherry-Tag Monoclonal (Elabscience) at a dilution of 1:1000 for SGR58. The secondary antibodies used were Horse anti-mouse IgG HRP (Cell Signalling Technology) at a dilution of 1:5000 for anti-GFP and 1:3000 for anti-mCherry-Tag Monoclonal, and Goat anti-Rat IgG (H+L) HRP (Thermo Fisher Scientific) 1:5000. Pierce ECL Western Blotting Substrate (Thermo Fisher Scientific) or SuperSignal West Femto chemiluminescent Substrate (Thermo Fisher Scientific) were used as detection agent.

Imaging of *C. elegans*

All microscopy imaging was carried out on a Zeiss M2 imager. Animals were mounted on 2.5% agar pads on glass slides. To immobilise, animals were picked into drop of 50mM NaN_3 (Thermo Fisher Scientific) on the pad. The NaN_3 was diluted from a 100mM stock using M9. Animals picked into the drop were left for 3-4 minutes before imaging.

Measurement of mCherry nuclear:cytoplasmic ratio

Measurement of mCherry nuclear/cytoplasmic ratio in animals expressing photocaged nanobody variants was performed using ImageJ software. Nuclear average brightness was measured in a region of interest within the nucleus avoiding interference from the nucleolus, from which nanobody::mCherry fusions appeared excluded. Cytoplasmic average was measured from the cytoplasmic area proximal to the nuclei, as per the method described by Kelley and Paschal.[5] The ImageJ “threshold” function was used to generate regions of interest encompassing the nuclei in the GFP channel. These “small” ROIs were used to generate a mask which was then enlarged by applying the ImageJ "dilate" function three times to generate the "large" ROIs which extended beyond the nucleus. The area and average brightness of these “small” and “large” ROIs were measured, and cytoplasmic brightness was acquired by the following formulae:

$$Cytoplasmic\ Total\ Intensity\ = \{(Large\ ROI\ Mean\ Intensity) \times (Large\ ROI\ Area)\}$$

$$- \{(Small\ ROI\ Mean\ Intensity) \times (Small\ ROI\ Area)\}$$

$$Cytoplasmic\ Area = Large\ ROI\ Area - Small\ ROI\ Area$$

$$Cytoplasmic\ Mean\ Intensity = \frac{Cytoplasmic\ Total\ Intensity}{Cytoplasmic\ Area}$$

Statistical analysis of mCherry nuclear:cytoplasmic ratio measurements

Statistical significance of subcellular localisation results was determined using a two-tailed Welch's T-test. Regressions were fit using a one-phase linear decay interpolation. Calculations were performed using GraphPad Prism version 9.0.2 for Windows (GraphPad Software).

Parameterisation of photocaged amino acids for molecular dynamics

To prepare input files for MD simulations of $eNB^{Y37ONBY}$ and eNB^{Y37NPY} it was necessary to generate parameters for the ONBY and NPY caging groups. These parameters were derived from the Generalised Amber Force Field (GAFF)[6] and supplemented with quantum mechanical parameters for the nitro-group.[7] A detailed description of the process, including files, code, and Python environment used to generate the input files for these simulations, can be found at https://github.com/wells-wood-research/oshea-j-interface-engineering-2021.

Molecular dynamics of nanobody/GFP complexes

Files were prepared for molecular dynamics using AmberTools18[6] and Open Babel.[8] Molecular dynamics simulations were performed using OpenMM.[9] The non-bonding interactions were modelled by PME, and the cut-off for non-bonding interactions was 1nm. Bonds involving hydrogen atoms were constrained in length. Simulations were run at 1bar and 300K. Pressure was maintained by a Monte Carlo barostat and temperature was maintained by the Langevin integrator. The frictional constant for the Langevin integrator was $1ps^{-1}$. A timestep of 2fs was used. A detailed description of the simulations, including the files, code, and Python environment used to perform them, can be found at https://github.com/wells-wood-research/oshea-j-interface-engineering-2021.

Alanine scanning of nanobody/GFP complexes

Alanine scanning was performed using the open-source program BUDE Alanine Scan. A detailed description of the process, including the environment, files, and code used, can be found at https://github.com/wells-wood-research/oshea-j-interface-engineering-2021.

Cloning plasmids for *E.coli* periplasmic protein expression

For expression of proteins in the periplasm of *E. coli*, constructs were cloned into the pSANG10-3F plasmid, and these plasmids were transformed into BL21(DE3) cells (New England Biolabs).

Expression and purification of GST::GFP and GST

Plasmids for expression of GST::GFP or GST were transformed into BL21(DE3) cells (New England Biolabs) under conditions specified by the manufacturer. The transformation was grown on TB agar plates supplemented with carbenicillin (200ug/mL) at 37°C overnight. The next day, a colony was picked to inoculate 3mL of TB supplemented with carbenicillin (200ug/mL) at 37°C with shaking at 220rpm overnight. The next day, this culture was used at 1000x to inoculate AIM-2YT Broth Base including trace elements (Formedium)

supplemented with carbenicillin (400ug/mL). For GST::GFP fusion or GST alone, cultures of 150mL were used. For nanobody mutants, cultures of 1L were used. The culture was grown at 37°C for 2 hours, then 30°C overnight, shaking at 220rpm.

For purification of GST::GFP fusion or GST alone, the culture was split between 10 15mL falcon tubes and cells were centrifuged (4000G. 4°C, 10 minutes). Pellets were stored at -20°C for up to 2 weeks. When protein was required, cells were resuspended in BugBuster Extraction Reagent (Merck Life Sciences) supplemented with lysozyme (25ng/mL, Thermo Fisher Scientific) cOmplete EDTA-free protease inhibitor cocktail tablets (1 tablet per 50mL, Merck Life Sciences), and DNaseI (2U/mL, New England Biolabs). Protein was recovered as per the method described for BugBuster protein extraction described by the manufacturer.

Expression and purification of nanobody mutants

Plasmids for expression of given protein was transformed into BL21(DE3) cells (New England Biolabs) under conditions specified by the manufacturer. The transformation was grown on TB agar plates supplemented with kanamycin (100ug/mL) at 37°C overnight. The next day, a colony was picked to inoculate 3mL of TB supplemented with kanamycin (100ug/mL) at 37°C with shaking at rpm overnight. The next day, this culture was used at 1000x to inoculate 1L AIM-2YT Broth Base including trace elements (Foremedium) supplemented with kanamycin (100ug/mL). The culture was grown at 37°C for 2 hours shaking at 220rpm, then 30°C overnight shaking at 160rpm.

Cultures were centrifuged (4000G, 4°C, 10 minutes). The supernatant was discarded. The cells were resuspended in 5mL lysis buffer per gram of pellet. The lysis buffer consisted of BugBuster Extraction Reagent (Merck Life Sciences) supplemented with lysozyme (25ng/mL, Thermo Fisher Scientific), cOmplete EDTA-free protease inhibitor tablets (1 tablet per 50mL, Merck Life Sciences), and DNaseI (2U/mL. New England Biolabs). The lysis was incubated at room temperature for 30 minutes with gentle shaking, then centrifuged (16,000G, 4°C, 20 minutes). The supernatant was recovered at stored at 4°C. The supernatant was supplemented with 1mL Ni-NTA resin and 10mM imidazole, then incubated at 4°C while rolling for 1 hour. Ni-NTA beads were centrifuged briefly, the supernatant discarded, and the beads resuspended in 5mL of ice-cold wash buffer (20mM imidazole in 1xPBS). This wash was performed a total of 3 times. After the final wash, the beads were resuspended in 1mL of ice-cold elution buffer (500mM imidazole in 1xPBS), left gently rocking for 10 minutes at room temperature, then briefly centrifuged, supernatant recovered and stored at 4°C, and the beads resuspended again in 1mL elution buffer. This elution was performed a total of 4 times, and all recovered supernatants were assayed by SDS-PAGE. 5uL of sample was diluted 1:1 with a 4:1 mix of 4X Bolt LDS Sample Buffer (Life Technologies) and NuPAGE Sample Reducing Agent (Thermo Fisher Scientific) respectively. The mix was shaken at 95°C for 15 minutes to denature, then electrophoresis was performed on precast Bolt 4 to 12% gels (Thermo Fisher Scientific) for 18 min at 200V. The gel was incubated in Coomassie reagent shaking at room temperature for 15 minutes before imaging. The elute fractions with little contamination and significant amount of nanobody were combined, transferred into 12-14,000 Da MWCO dialysis tubing (Scientific Laboratories Supplies) and dialysed against 10L of 1xPBS at 4°C overnight, then 2L of 1xPBS at 4°C for 1 hour. The dialysed fraction was recovered, and presence of desired protein was determined by SDS-PAGE.

The dialysed fraction was concentrated using Amicon Ultra 0.5mL 3kDa Molecular Weight Cut Off columns (Merck Life Sciences) by centrifugation at 14,000 G. Protein concentration was determined by Bradford assay against a BSA standard.

ELISA of nanobody/GFP interactions

GST-containing protein (either GST::GFP for main experiments or GST alone for the non-specific binding control) was diluted in PBS to a final concentration of 3µg/mL. 100 µl of GST-containing protein was then added to wells of a Pierce™ glutathione coated white 96-well plate (Thermo Fisher Scientific), and allowed to bind for 1 hour at room temperature, with gentle shaking. For each nanobody concentration to be assayed 3 wells were prepared, as well as two wells for a negative control, without nanobody. Unbound GST-containing protein was removed from the wells by a single downward shaking motion, by hand. Any liquid remaining in the plate was then removed by striking the upturned plate onto tissue paper, 10 times. Each well was then washed with 100 µl of PBST, for 60 seconds. The PBST was removed from the wells as described above, and this wash was performed 4 times.

100 µl of diluted nanobody was then added to each well, diluted in PBS. Each nanobody concentration was assayed in duplicate during assay optimisation, and in triplicate for final measurements. Negative control wells were included in which only PBS was added. Nanobodies were allowed to bind to GFP for 1 hour at room temperature, with gentle shaking. Excess nanobody was then removed, and wells were washed 4 times as before.

100 µl of mouse anti-FLAG M2 antibody (Merck Life Sciences) was then added to the wells, diluted 1:1000 (v/v) in PBST-BSA (3%). This was allowed to bind for 1 hour at room temperature, with gentle shaking. Antibody was then removed, and wells washed as before.

100 µl of secondary HRP-conjugated rabbit anti-mouse P026002 antibody (Agilent) was then added, diluted 1:1000 (v/v) in PBST-BSA (3%). This was allowed to bind for 1 hour at room temperature, with gentle shaking. This was then removed and wells were washed, as before.

Following the final washes, 100 µl of ECL reagent (luminol) was added to each well, and the plate was then immediately scanned for luminescence using a Fluoroskan Ascent FL plate reader (Thermo Fisher Scientific).

The mean of the background luminescence was calculated from wells where PBS was added instead of nanobody, and then subtracted from each measured value to obtain background-adjusted luminescence. Due to variations in absolute luminescence values for different nanobodies, the values were then normalised vs the lowest and highest luminescence values for each ligand titration. Curves were then fit to the data using GraphPad Prism 9.1, using the 'One site – specific binding' pre-set, thus calculating affinity values and 95% CI.

Supplementary Tables

Supplementary Table 1 – Transgenic *C. elegans* Strains

Name	Description	Genetic background	Plasmid 1	Plasmid 2	Plasmid 3
SGR57	Psur-5::HuPKIαNES::MmNPYRS; Prpr-1::tRNA(C15); Prps-0::GFP(am)::mCherry::HA::EGL-13NLS	smg-6	JOS97	ZX297	SG88
SGR58	Psur-5::HuPKIαNES::MmNPYRS; Prpr-1::tRNA(C15); Psur-5::enhancer(Y37am)::mCherry::SL2::GFP::EGL-13NLS	N2	JOS97	ZX297	JOS94
SGR59	Psur-5::HuPKIαNES::MmNPYRS; Prpr-1::tRNA(C15); Psur-5::mCherry::SL2::GFP::EGL-13NLS	N2	JOS97	ZX297	JOS77
SGR60	Psur-5::HuPKIαNES::MmNPYRS; Prpr-1::tRNA(C15); Psur-5::enhancer::mCherry::SL2::GFP::EGL-13NLS	N2	JOS97	ZX297	JOS52
SGR61	Psur-5::HuPKIαNES::MmNPYRS; Prpr-1::tRNA(C15); Psur-5::enhancer::mCherry::SL2::EGL-13NLS::BFP	N2	JOS97	ZX297	JOS233
SGR62	Psur-5::HuPKIαNES::MmNPYRS; Prpr-1::tRNA(C15); Psur-5::enhancer(Y37am, R35A)::mCherry::SL2::GFP::EGL-13NLS	N2	JOS97	ZX297	JOS137
SGR63	Psur-5::HuPKIαNES::MmNPYRS; Prpr-1::tRNA(C15); Psur-5::enhancer(Y37am, E103A)::mCherry::SL2::GFP::EGL-13NLS	N2	JOS97	ZX297	JOS145
SGR64	Psur-5::HuPKIαNES::MmNPYRS; Prpr-1::tRNA(C15); Psur-5::enhancer(Y37am, R35A, E103A)::mCherry::SL2::GFP::EGL-13NLS	N2	JOS97	ZX297	JOS140
SGR65	Psur-5::HuPKIαNES::MmNPYRS; Prpr-1::tRNA(C15); Psur-5::enhancer(Y37am, W47A)::mCherry::SL2::GFP::EGL-13NLS	N2	JOS97	ZX297	JOS142
SGR66	Psur-5::HuPKIαNES::MmNPYRS; Prpr-1::tRNA(C15); Psur-5::minimiser(Y116am)::mCherry::HA::SL2::GFP::EGL-13NLS	smg-6	JOS97	SE150	JOS229
SGR67	Psur-5::HuPKIαNES::MmNPYRS; Prpr-1::tRNA(C15); Psur-5::minimiser(Y116am, D98A)::mCherry::HA::SL2::GFP::EGL-13NLS	smg-6	JOS97	SE150	JOS230
SGR68	Psur-5::HuPKIαNES::MmNPYRS; Prpr-1::tRNA(C15); Psur-5::minimiser(Y116am, D119A)::mCherry::HA::SL2::GFP::EGL-13NLS	smg-6	JOS97	SE150	JOS232
SGR96	Psur-5::HuPKIαNES::MmNPYRS; Prpr-1::tRNA(C15); Psur-5::enhancer(R35A)::mCherry::SL2::GFP::EGL-13NLS	N2	JOS97	SE150	JOS287
SGR97	Psur-5::HuPKIαNES::MmNPYRS; Prpr-1::tRNA(C15); Psur-5::enhancer(E103A)::mCherry::SL2::GFP::EGL-13NLS	N2	JOS97	SE150	JOS288
SGR98	Psur-5::HuPKIαNES::MmNPYRS; Prpr-1::tRNA(C15); Psur-5::enhancer(W47A)::mCherry::SL2::GFP::EGL-13NLS	N2	JOS97	SE150	JOS289
SGR99	Psur-5::HuPKIαNES::MmNPYRS; Prpr-1::tRNA(C15); Psur-5::enhancer(R35A, E103A)::mCherry::SL2::GFP::EGL-13NLS	N2	JOS97	SE150	JOS290

Supplementary Table 2 – Expression plasmids

Name	Description	Source plasmids			
		Destination	P4-P1r	221	P2r-P3
JOS97	Psur-5::HuPKIαNES::MmNPYRS	IR98	SE72	KB125	SG606
JOS77	Psur-5::mCherry::SL2::GFP::EGL-13NLS	pDEST	SE72	IR186	LD343
JOS233	Psur-5::enhancer::mCherry::SL2::EGL-13NLS::BFP	JOS163	SE72	JOS4	JOS164
JOS137	Psur-5::enhancer(Y37am, R35A)::mCherry::SL2::GFP::EGL-13NLS	JOS13	SE72	JOS117	JOS19
JOS145	Psur-5::enhancer(Y37am, E101A)::mCherry::SL2::GFP::EGL-13NLS	JOS13	SE72	JOS125	JOS19
JOS94	Psur-5::enhancer(Y37am)::mCherry::SL2::GFP::EGL-13NLS	JOS13	SE72	JOS41	JOS6
JOS52	Psur-5::enhancer::mCherry::SL2::GFP::EGL-13NLS	JOS13	SE72	JOS4	JOS6
JOS140	Psur-5::enhancer(Y37am, R35A, E103A)::mCherry::SL2::GFP::EGL-13NLS	JOS13	SE72	JOS120	JOS19
JOS142	Psur-5::enhancer(Y37am, W47A)::mCherry::SL2::GFP::EGL-13NLS	JOS13	SE72	JOS122	JOS19
SG88	Prps-0::GFP(am)::mCherry::HA::EGL-13NLS	[10]			
JOS229	Psur-5::minimiser(Y116am)::mCherry::HA::SL2::GFP::EGL-13NLS	JOS13	SE72	JOS40	JOS164
JOS230	Psur-5::minimiser(Y116am, D98A)::mCherry::HA::SL2::GFP::EGL-13NLS	JOS13	SE72	JOS180	JOS164
JOS232	Psur-5::minimiser(Y116am, D119A)::mCherry::HA::SL2::GFP::EGL-13NLS	JOS13	SE72	JOS182	JOS164

JOS287	Psur-5::enhancer(Y37am, R35A)::mCherry::SL2::GFP::EGL-13NLS	JOS13	SE72	JOS165	JOS164
JOS288	Psur-5::enhancer(Y37am, E101A)::mCherry::SL2::GFP::EGL-13NLS	JOS13	SE72	JOS167	JOS164
JOS288	Psur-5::enhancer(Y37am, W47A)::mCherry::SL2::GFP::EGL-13NLS	JOS13	SE72	JOS240	JOS164
JOS290	Psur-5::enhancer(Y37am, R35A, E101A)::mCherry::SL2::GFP::EGL-13NLS	JOS13	SE72	JOS239	JOS164

Supplementary Table 3.1 – Destination Vectors

Name	Description	Source
IR98	pDEST R4-R3 Prps-0::HygR	[4]
JOS13	pDEST R4-R3 SL2::GFP-NLS::let-858 3' UTR	Made from LD343 and SE114. SL2::GFP-NLS-let858 was amplified from LD343. SE114 was digested with BglII and the amplified construct was assembled with the larger digestion product using NEBuilder.<br>Primers used for amplification:<br>J92 - gibson::SL2 F:<br>CATTTTACGTTTCTCGTTCAACTTTATTATACATAGTTGAgatctAGATCTGCTGTCTCATCCTACTTTCAC<br>J93 - let858::gibson R:<br>TCCCAGTCACGACGTTGTAAAACGACGGCCAGTGAATTAagatcTagatcTATACGGATTCGCATTTGCCA
SE114	pDEST R4-R3 II BglII::let-858 3' UTR::BglII	Made from SG606 and pDESTII R3-R4. pDESTII R3-R4 was opened by PCR amplification. let-858 was amplified from SG606. Both amplified products were digested with BglII, then ligated together.<br>Primers for pDESTII R3-R4 amplification:<br>336: CATAGTTGAgatctTAATTCACTGGCCGTCGTTTTACAAC<br>337: TGAATTAagatcTCAACTATGTATAATAAAGTTGAACGAGAAACG<br>Primers for SG606 amplification:<br>339:<br>CATTTTACGTTTCTCGTTCAACTTTATTATACATAGTTGAgatctCGTGAAGTGGAATCGGATGATC<br>340:<br>TCCCAGTCACGACGTTGTAAAACGACGGCCAGTGAATTAagatcTATACGGATTCGCATTTGCCAAG
SG72	pDEST R4-R3 SL2::GFP::let-858 3' UTR	[10]
JOS163	pDEST R4-R3 SL2::EGL13NLS::mTagBFP2::let-858 3' UTR	Made from JOS13 and gBlock gJ3. JOS13 was opened by PCR. The amplified product was recovered and assembled with gJ3 by NEBuilder.<br>Primers for JOS13 opening:<br>582 - let858 F:<br>TAACGTGAAGTGGAATCGGATGATC<br>579 - SL2 R:<br>TTTTTCTACCGGTACAGCAGTTTCC

Supplementary Table 3.2 – pENTR P4-P1r Vectors

Name	Description	Source
SE72	Psur-5	Amplified from genonimic DNA.<br>189 P sur-5s attB4F:<br>GGGGACAACTTTGTATAGAAAAGTTGCGCAGGCGGTAAACATACGTTG<br>193 P sur-5 attB1R:<br>GGGGACTGCTTTTTTGTACAAACTTGTCTGAAAACAAAATGTAAAGTTCAAAGG

Supplementary Table 3.3 – pENTR 221 Vectors

Name	Description	Source
ZX297	Prpr-1::tRNA(C15)::sup-7 short 4x	Made from ZX296. ZX296 was digested with SalI and BamHI and the fragment containing two "Prpr-1 Bt mttRNA Ser C15 sup-7" repeats was recovered. ZX296 was opened by digest with SalI and BglII. These two digest products were ligated together.
ZX296	Prpr-1::tRNA(C15)::up-7 short 2x	Made from SE150. SE150 was digested with SalI and BamHI and the Prpr-1 Bt mttRNA Ser C15 sup-7 fragment was recovered. SE150 was opened by digest with SalI and BglII. These two digest products were ligated together.

SE150	Prpr-1::mttRNA(C15)::sup-7 short	PylT in SG322 was replaced with Bt mttRNA C15 Sequence: GGAAACCTGgTCAgGgAGAcCGAAcGGACTCTAAATCCGTTCAGCCGGGTTcGATTCCCGGGGTTTCCG
JOS4	enhancer::HL	G-block gJ1 was recombined into pDONR 221 by BP recombinase.
JOS5	minimiser::HL	G-block gJ2 was recombined into pDONR 221 by BP recombinase.
JOS41	enhancer(Y37am)::HL	Made from JOS4. JOS4 was amplified with primers that applied an amber stop codon mutation to Y37. The amplified product was recovered and circularised by NEBuilder.<br>J14 - enhY37 F: CGTTACTCCATGCGTTGGTAGCGTCAAGCCCCAGGAAAGG<br>J15 - enhY37 R: CCTTTCCTGGGGCTTGACGCTACCAACGCATGGAGTAACG
JOS117	enhancer(Y37am, R35A)::HL	Made from JOS41. JOS41 was amplified with primers that applied the mutation R35A. The amplified product was recovered and circularised by NEBuilder.<br>J167 - Cele enhancer(Y37am) R35A F: TTACTCCATGGCTTGGTAGCGTC<br>J168 - Cele enhancer(Y37am) R35A R: GACGCTACCAAGCCATGGAGTAA
JOS125	enhancer(Y37am, E103A)::HL	Made from JOS41. JOS41 was amplified with primers that applied the mutation E103A. The amplified product was recovered and circularised by NEBuilder.<br>J175 - Cele enhancer E103A F: CGTCGGATTCGCTTACTGGGGAC<br>J176 - Cele enhancer E103A R: GTCCCCAGTAAGCGAATCCGACG
JOS120	enhancer(Y37am, R35A, E103A)::HL	Made from JOS117. JOS117 was amplified with primers that applied the mutation E103A. The amplified product was recovered and circularised by NEBuilder.<br>J175 - Cele enhancer E103A F: CGTCGGATTCGCTTACTGGGGAC<br>J176 - Cele enhancer E103A R: GTCCCCAGTAAGCGAATCCGACG
JOS122	enhancer(Y37am, W47A)::HL	Made from JOS41. JOS41 was amplified with primers that applied the mutation W47A. The amplified product was recovered and circularised by NEBuilder.<br>J171 - Cele enhancer W47A F: GGAGCGTGAGGCTGTCGCCGGAA<br>J172 - Cele enhancer W47A R: TTCCGGCGACAGCCTCACGCTCC
JOS40	minimiser(Y116am)::HL	Made from JOS5. JOS5 was amplified with primers that applied an amber stop codon mutation to Y116. The amplified product was recovered and circularised by NEBuilder<br>J10 - minY100B F: CTCTCCTGCCTCGACTAGGTCATGGACTACTGGGG<br>J11 - minY100B R: CCCCAGTAGTCCATGACCTAGTCGAGGCAGGAGAG
JOS180	minimiser(Y116am, D98A)::HL	Made from JOS40. JOS40 was amplified with primers that applied the mutation D98A. The amplified product was recovered and circularised by NEBuilder.<br>J255 - Cele minimiser D95 F: CGCCGCCGCTTCCGGAACCCAACTCGGATACGTCGGAGCCG<br>J244 - Cele minimiser(Y100Bam) D95 R: TTCCGGAAGCGGCGGCGCAGTAGTAACGGGCGG
JOS181	minimise(Y116am, D119A)::HL	Made from JOS40. JOS40 was amplified with primers that applied the mutation D119A. The amplified product was recovered and circularised by NEBuilder.<br>J256 - Cele minimser(Y100Mam) D101A F: CTAGGTCATGGCTTACTGGGGAAAGGGAACCCAAGTCACCG<br>J257 - Cele minimser(Y100Mam) D101A R: TTCCCCAGTAAGCCATGACCTAGTCGAGGCAGGAGAGTCCGACG
KB125	HuPKIαNES::MmNPYRS	Made from LD184. LD184 was opened at the 5' end of the synthetase by PCR, then a primer containing the Hu PKIa NES incorporated at the opening by NEBuilder.<br>Plasmids for amplification:<br>431 MmPylRS NEB R: CATTTTTGCAGCCTGCTTTTTTGTACA<br>430 MmPylRS NEB F: ATGGACAAGAAGCCACTCAACAC<br>Plasmid for NEBuilder:<br>AAAAGCAGGCTGCAAAAATGCTCGCCCTCAAGCTCGCCGGACTCGACATCATGGACAAGAAGCCACTCAA
LD184	MmNPYRS	G-block gL was recombined into pDONR221 by BP recombinase.
SG322	Prpr-1::PylT::sup-7	P*rpr-1* was amplified from genomic DNA and fused to PylT by overlap extension PCR, 100bp of the sup-7 3' region was also fused to the 3' end of PylT

		by overlap extension PCR
JOS165	enhancer(R35A)::HL	Made from JOS117. JOS117 was amplified with primers that applied the am37Y mutation, reverting the amber mutation at position 37 back to its wildtype tyrosine codon. The amplified product was recovered and circularised by NEBuilder.<br>J285 - Cele enhancer(R35A) am37Y F:<br>TGGCTTGGTACCGTCAAGCCCCAGGAAAGGAGCGTGAGTGG<br>J286 - cele enhancer(R35A) am37Y R:<br>GCTTGACGGTACCAAGCCATGGAGTAACGGTTGACTGGGAATCCG
JOS167	enhancer(E103A)::HL	Made from JOS125. JOS125 was amplified with primers that applied the am37Y mutation, reverting the amber mutation at position 37 back to its wildtype tyrosine codon. The amplified product was recovered and circularised by NEBuilder.<br>J276 - Cele enhancer am37Y F:<br>GCGTTGGTACCGTCAAGCCCCAGGAAAGGAGCG<br>J277 - v2 Cele enhancer am37Y R:<br>CTTGACGGTACCAACGCATGGAGTAACGGTTGACTGG
JOS240	enhancer(W47A)::HL	Made from JOS122. JOS122 was amplified with primers that applied the am37Y mutation, reverting the amber mutation at position 37 back to its wildtype tyrosine codon. The amplified product was recovered and circularised by NEBuilder.<br>J276 - Cele enhancer am37Y F:<br>GCGTTGGTACCGTCAAGCCCCAGGAAAGGAGCG<br>J277 - v2 Cele enhancer am37Y R:<br>CTTGACGGTACCAACGCATGGAGTAACGGTTGACTGG
JOS239	enhancer(R35A, E103A)::HL	Made from JOS120. JOS120 was amplified with primers that applied the am37Y mutation, reverting the amber mutation at position 37 back to its wildtype tyrosine codon. The amplified product was recovered and circularised by NEBuilder.<br>J285 - Cele enhancer(R35A) am37Y F:<br>TGGCTTGGTACCGTCAAGCCCCAGGAAAGGAGCGTGAGTGG<br>J286 - cele enhancer(R35A) am37Y R:<br>GCTTGACGGTACCAAGCCATGGAGTAACGGTTGACTGGGAATCCG

Supplementary Table 3.4 – pENTR P2r-P3 Vectors

Name	Description	Source
LD343	SL2::GFP-NLS::let-858 3’UTR	Made from IR182. NLS-egl13 was attached to the 3' end of GFP, and flanking BP recombinase binding sites were added for recombination into pDONR P2r-P3 vector<br>Plasmids for amplification of fragment 1:<br>140 attB2R 5' SL2 GFP F:<br>GGGGACAGCTTTCTTGTACAAAGTGGgctgtctcatcctactttcacctagttaa<br>214 GFP NLS R:<br>TTCGCGTTTTCACTCAGTTTTGTCGGATTCGCTTTTCGTCTACGGCTCATtcctcccttg tagagctcgtccattccg<br>Plasmids for amplification of fragment 2:<br>213 GFP NLS F:<br>TCCGACAAAACTGAGTGAAAACGCGAAGAAGCTTGCCAAGGAAGTTGAAAATtaa cgtgaagtggaatcggatgatcgacgccg<br>145 attB3 3' GFP let858 R:<br>GGGGACAACTTTGTATAATAAAGTTGatacggattcgcatttgccaag
SG606	let-858 3'UTR	PCR amplified from genomic DNA<br>Plasmids for amplification:<br>725 let-858 3’ attB2R:<br>GGGGACCACTTTGTACAAGAAAGCTGGGTATACGGATTCGCATTTGCCAAGC<br>726 let-858 3’ attB3R:<br>GGGGACAACTTTGTATAATAAAGTTGATACGGATTCGCATTTGCCAAGC
JOS6	mCherry	Made from SG79. mCherry was amplified from SG79 and flanking BP recombinase binding sites were added for recombination into pDONR P2r-P3.<br>J30 - attR2::mCherry:<br>GGGGACAGCTTTCTTGTACAAAGTGGTAGTCTCAAAGGGTGAAGAAGATAACAT GG<br>J31 - mCherry::attL3 R:<br>GGGGACAACTTTGTATAATAAAGTTGTTTACTTATACAATTCATCCATGCCACCTG T

JOS19	mCherry::HisTagx6	Made from JOS6. mCherry was amplified from JOS6 and an N-terminal 6x His repeat was added. Flanking BP recombinase binding sites were added for recombination into pDONR P2r-P3.<br>J30 - attR2::mCherry:<br>GGGGACAGCTTTCTTGTACAAAGTGGTAGTCTCAAAGGGTGAAGAAGATAACATGG<br>J141 - mCherry::6xHis::attL2 R:<br>GGGGACAACTTTGTATAATAAAGTTGTTTAATGATGATGATGATGATGCTTATACAATTCATCCATGCCACCTGT
JOS164	mCherry::HisTagx6::HA	Made from JOS19. mCherry::HisTagx6 was amplified from JOS19 and an N-terminal HA tag was added. Flanking BP recombinase binding sites were added for recombination into pDONR P2r-P3.<br>J30 - attR2::mCherry:<br>GGGGACAGCTTTCTTGTACAAAGTGGTAGTCTCAAAGGGTGAAGAAGATAACATGG<br>J234 - mCherry::His-tag::HA R:<br>CGTAGTCTGGGACGTCGTATGGGTAATGATGATGATGATGATGCTTATACAATTCATCC
SG79	mCherry::HA	[10]
IR182	SL2::GFP ::let-858 3' UTR	[11]

Supplementary Table 4 – G-blocks

Name	Description	Sequence
gJ1	attB1::enhancer::HL::attB2<br><br>*C. elegans* optimised enhancer nanobody and glycine-rich linker	ACAAGTTTGTACAAAAAAGCAGGCTaaaaATGGCCCAAGTCCAACTCGTCGAGTCCGGAGGAGCCCTCGTCCAACCAGGAGGATCCCTCCGTCTCTCCTGCGCCGCCTCCGGATTCCCAGTCAACCGTTACTCCATGCGTTGGTACCGTCAAGCCCCAGGAAAGGAGCGTGAGTGGGTCGCCGGAATGTCCTCCGCCGGAGACCGTTCCTCCTACGAGGACTCCGTCAAGGGACGTTTCACCATCTCCCGTGACGACGCCCGTAACACCGTCTACCTCCAAATGAACTCCCTCAAGCCAGAGGACACCGCCGTCTACTACTGCAACGTCAACGTCGGATTCGAGTACTGGGGACAAGGAACCCAAGTCACCGTCTCCTCCATCCTCGGAGCCCCATCCGGAGGAGGAGCCACCGCCGGAGCCGGAGGAGCCGGAGGACCAGCCGGACTCATCCACCCAGCTTTCTTGTACAAAGTGGT
gJ2	attB1::minimiser::HL::attB2<br><br>*C. elegans* optimised minimiser nanobody and glycine-rich linker	ACAAGTTTGTACAAAAAAGCAGGCTaaaaATGGCCGTCCAACTCCAAGAGTCCGGAGGAGGATCCGTCCAAGCCGGAGGATCCCTCCGTCTCTCCTGCGCCGCCTCCGGAGACACCTTCTCCTCCTACTCCATGGCCTGGTTCCGTCAAGCCCCAGGAAAGGAGTGCGAGCTCGTCTCCAACATCCTCCGTGACGGAACCACCACCTACGCCGGATCCGTCAAGGGACGTTTCACCATCTCCCGTGACGACGCCAAGAACACCGTCTACCTCCAAATGGTCAACCTCAAGTCCGAGGACACCGCCCGTTACTACTGCGCCGCCGACTCCGGAACCCAACTCGGATACGTCGGAGCCGTCGGACTCTCCTGCCTCGACTACGTCATGGACTACTGGGGAAAGGGAACCCAAGTCACCGTCTCCATCCTCGGAGCCCCATCCGGAGGAGGAGCCACCGCCGGAGCCGGAGGAGCCGGAGGACCAGCCGGACTCATCTACCCAGCTTTCTTGTACAAAGTGGT
gJ3	EGL-13 NLS::mTagBFP2<br><br>*C. elegans* optimised mTagBFP with 5' SL2 overlap and EGL-13 NLS	GGAAACTGCTGTACCGGTAGAAAAAATGAGCCGAAGACGAAAAGCCAACCCAACTAAACTGTCTGAGAACGCCAAGAAGCTCGCCAAGGAGGTCGAGAACATGGTCTCCAAGGGAGAGGAGCTCATCAAGGAGAACATGCACATGAAGCTCTACATGGAGGGAACCGTCGACAACCACCACTTCAAGTGCACCTCCGAGGGAGAGGGAAAGCCATACGAGGGAACCCAAACCATGCGTATCAAGGTCGTCGAGGGAGGACCACTCCCATTCGCCTTCGACATCCTCGCCACCTCCTTCCTCTACGGATCCAAGgtaagtttaaacatatatatactaactaaccctgattatttaaattttcagACCTTCATCAACCACACCCAAGGAATCCCAGACTTCTTCAAGCAATCCTTCCCAGAGGGATTCACCTGGGAGCGTGTCACCACCTACGAGGACGGAGGAGTCCTCACCGCCACCCAAGACACCTCCCTCCAAGACGGATGCCTCATCTACAACGTCAAGgtaagtttaaacagttcggtactaactaaccatacatatttaaattttcagATCCGTGGAGTCAACTTCACCTCCAACGGACCAGTCATGCAAAAGAAGACCCTCGGATGGGAGGCCTTCACCGAGACCCTCTACCCAGCCGACGGAGGACTCGAGGGACGTAACGACATGGCCCTCAAGCTCGTCGGAGGATCCCACCTCATCGCCAACGCCAAGgtaagtttaaacatgattttactaactaactaatctgatttaaattttcagACCACCTACCGTTCCAAGAAGCCAGCCAAGAACCTCAAGATGCCAGGAGTCTACTACGTCGACTACCGTCTCGAGCGTATCAAGGAGGCCAACAACGAGACCTACGTCGAGCAACACGAGGTCGCCGTCGCCCGTTACTGCGACCTCCCATCCAAGCTCGGACACAAGCTCAACTAACGTGAAGTGGAATCGGATGATC
gJ4	Enhancer(*E. coli*)::HL::mKate<br><br>*E. coli* optimised enhancer nanobody, glycine rich linker, mKate	GGGCTAACAGGAGGAATTAACCATGGCTCAAGTTCAGCTGGTTGAGTCGGGCGGCGCCCTTGTTCAACCTGGTGGTAGCCTGCGGTTGTCTTGCGCCGCAAGTGGGTTCCCAGTCAACCGGTATTCGATGAGATGGTACCGGCAGGCGCCCGGTAAAGAGAGAGAATGGGTCGCCGGAATGTCTTCCGCGGGCGATCGCTCTAGTTATGAGGATTCCGTGAAGGGCCGCTTCACAATCTCCCGCGACGATGCACGTAACACTGTGTATTTGCAGATGAACAGCTTAAAGCCTGAGGACACAGCCGTTTATTACTGCAATGTCAATGTCGGTTTTGAATATTGGGGCCAGGGAACTCAGGTCACTGTTTCATCTGGATCCATCCTGGGTGCACCCTCTGGAGGGGGCGCCACTAGCACTGCAGGAGCCGGGGGAGCAGGAGGACCAGCTGGACTGATCAGCGAACTGATTAAGGAGAATATGCACATGAAACTTTACATGGAAGGTACGGTTAACAACCATCACTTTAAATGCACCTCTGAAGGCGAGGGTAAGCCCTACGAAGGCACTCAGACTATGCGGATCAAAGCTGTTGAAGGAGGCCCTTTACCATTCGCGTTTGACATCTTAGCAACATCCTTTATGTACGGAAGTAAGACGTTTATCAATCATACG

		CAAGGGATTCCCGATTTTTTTAAGCAATCGTTCCCCGAAGGCTTCACTTGGGAGAGAGTCACGACGTA<br>CGAGGACGGGGGCGTGTTAACCGCAACACAAGACACATCCCTGCAAGATGGCTGTCTTATTTACAAT<br>GTAAAAATTAGAGGTGTAAATTTTCCCAGCAACGGCCCAGTGATGCAAAAGAAAACATTGGGATGGG<br>AGGCTAGCACTGAGACGCTTTACCCTGCGGATGGCGGATTAGAAGGACGCGCCGACATGGCACTTAA<br>GCTTGTGGGAGGGGGACATTTAATATGCAATCTGAAGACAACTTACCGCAGTAAGAAACCCGCTAAA<br>AATCTTAAGATGCCCGGGGTATATTACGTCGACCGTCGGCTGGAGCGTATAAAAGAGGCTGATAAGG<br>AGACTTACGTTGAGCAGCATGAAGTAGCTGTCGCCCGCTATTGCGATCTTCCTTCGAAGTTGGGCCAC<br>CGCCATCATCATCATCACCACTAAAGCTCGAGCGAAgcttgggcccgaacaaaa
gJ5	Mb NPYRS(*E. coli*) mutations<br><br>*E. coli* section of the *Methanosarcina barkeri* pyrrolysine aminoacyl-tRNA synthetase mutations with the mutations necessary for binding photocaged tyrosines	gtccgatgctggccccgaCCtttTATAACTATatgCGTAAACTGGATCGTATTCTGCCGGGTCCGATCAAAATTT<br>TTGAAGTGGGCCCGTGCTATCGCAAAGAAAGCGATGGCAAAGAACACCTGGAAGAATTCACCATGGT<br>TggcTTTggcCAAATGGGCAGCGGCTGCACCCGTGAAAACCTGGAAGCGCTGATCAAAGAATTCCTGG<br>ATTATCTGGAAATCGACTTCGAAATTGTGGGCGATAGCTGcatggtgtttggcgataccctggatattatGcaaatg<br>ggca
gL	attB1::MmNPYRS::attB2<br><br>*Methanosarcina mazei* pyrrolysine aminoacyl tRNA synthetase with mutations for binding photocaged tyrosine, codon optimised for expression in *C. elegans*.	GGGGACAAGTTTGTACAAAAAAGCAGGCTATGGACTACAAGGACGACGACGACAAGATGGACAAGA<br>AGCCACTCAACACCCTCATCTCCGCCACCGGACTCTGGATGTCCCGTACCGGAACCATCCACAAGATCA<br>AGCACCACGAGGTCTCCCGTTCCAAGATCTACATCGAGATGGCCTGCGGAGACCACCTCGTCGTCAAC<br>AACTCCCGTTCCTCCCGTACCGCCCGTGCCCTCCGTCACCACAAGTACCGTAAGACCTGCAAGCGTTGC<br>CGTGTCTCCGACGAGGACCTCAACAAGTTCCTCACCAAGGCCAACGAGGACCAAACCTCCGTCAAGGT<br>CAAGGTCGTCTCCGCCCCAACCCGTACCAAGAAGGTAAGTTTAAACATATcTATACTAACTAACCCTGA<br>TTATTTAAATTTTCAGGCCATGCCAAAGTCCGTCGCCCGTGCCCCAAAGCCACTCGAGAACACCGAGG<br>CCGCCCAAGCCCAACCATCCGGATCCAAGTTCTCCCCAGCCATCCCAGTCTCCACCCAAGAGTCCGTCT<br>CCGTCCCAGCCTCCGTCTCCACCTCCATCTCCTCCATCTCCACCGGAGCCACCGCCTCCGCCCTCGTCAA<br>GGGAAACACCAACCCAATCACCTCCATGTCCGCCCCAGTCCAAGCCTCCGCCCCAGCCCTCACCAAGTC<br>CCAAACCGACCGTCTCGAGGTCCTCCTCAACCCAAAGGACGAGATCTCCCTCAACTCCGGAAAGCCAT<br>TCCGTGAGCTCGAGTCCGAGCTCCTCTCCCGTCGTAAGGTAAGTTTAAACAGTTCGGTACTAACTAACC<br>ATACATATTTAAATTTTCAGAAGGACCTCCAACAAATCTACGCCGAGGAGCGTGAGAACTACCTCGGA<br>AAGCTCGAGCGTGAGATCACCCGTTTCTTCGTCGACCGTGGATTCCTCGAGATCAAGTCCCCAATCCTC<br>ATCCCACTCGAGTACATCGAGCGTATGGGAATCGACAACGACACCGAGCTCTCCAAGCAAATCTTCCG<br>TGTCGACAAGAACTTCTGCCTCCGTCCAATGCTCGCCCCAAACttcTACAACTACatgCGTAAGCTCGACC<br>GTGCCCTCCCAGACCCAATCAAGATCTTCGAGATCGGACCATGCTACCGTAAGGAGTCCGACGGAAA<br>GGTAAGTTTAAACATGATTTTACTAACTAACTAATCTGATTTAAATTTTCAGGAGCACCTCGAGGAGTT<br>CACCATGCTCggaTTCggaCAAATGGGATCCGGATGCACCCGTGAGAACCTCGAGTCCATCATCACCGA<br>CTTCCTCAACCACCTCGGAATCGACTTCAAGATCGTCGGAGACTCCTGCATGGTCttcGGAGACACCCT<br>CGACGTCATGCACGGAGACCTCGAGCTCTCCTCCGCCGTCGTCGGACCAATCCCACTCGACCGTGAGT<br>GGGGAATCGACAAGCCATGGATCGGAGCCGGATTCGGACTCGAGCGTCTCCTCAAGGTCAAGCACGA<br>CTTCAAGAACATCAAGCGTGCCGCCCGTTCCGAGTCCTACTACAACGGAATCTCCACCAACCTCTAAAC<br>CCAGCTTTCTTGTACAAAGTGGTCCCC

Supplementary Table 5 – Bacterial Expression plasmids

Name	Description	Source
pSANG 10-3F	Periplasmic expression construct	[12]
pULTR A-CNF	PylRS::PylRNA	Purchased from Addgene [13]
JOS103	Mb PylS NPYRS	*E. coli* optimised amino-acyl synthetase with mutations identified by *ref* to bind photocaged tyrosines. Cloned from gJ5.
SE381	Mb NPYRS::PylRNA::pUltra	pULTRA was digested with AccI and DraI. JOS103 was amplified with Seb primers 760, 761. These fragments were assembled using NEBuilder.<br>Seb 760 Mb NPYRS Ec F: tcgtaaaaacgattttcagcgcctg<br>Seb 761 Mb NPYRS Ec R:<br>aacagccaagctggagaccgtttaaacgcggccgcttacaggttcgtgctaatgcc
SE398	Mb NPYRS::Pyl-opt-tRNA::pUltra	SE381 was amplified with primers to introduce the optimised tRNA.<br>Seb 778 Pyl-opt F:<br>tggactctaaatccgttcagTGggttagattcccCACgtttccgccaaattcgaaaagc

		Seb 779 Pyl-opt R: ctgaacggatttagagtccattcgatctacatgatcaCgtttccaatgcggggcgcatc
pGEX-6P-1	GST::HRV 3C	Gift from Kathrin Ball lab.
JOS306	GST::HRV 3C::TEV::GFP	TEV::GFP cloned into pGEX-6P-1
JOS104	eNB::HL::mKate::His	Enhancer nanobody fused with mKate from gBlock gJ4 cloned into arabinose-inducible backbone.
JOS170	eNB(E103A)::HL::mKate::His	JOS104 was amplified using primers to introduce E103A mutation.<br>J161 - Ecoli enhancer E103A F: TGTCGGTTTTGCGTATTGGGGCC<br>J162 - Ecoli enhancer E103A R: GGCCCCAATACGCAAAACCGACA
JOS168	eNB(R35A)::HL::mKate::His	JOS107 was amplified using primers to revert position 37 from an amber stop codon to tyrosine.<br>J278 - Ecoli enhancer(R35A) am37Y F: TGGCGTGGTACCGGCAGGCGCCCGGTAAAGAGAGAGAATGG<br>J279 - Ecoli enhancer(R35A) am37Y R: GCCTGCCGGTACCACGCCATCGAATACCGGTTGACTGGGAACCCACTTGCG
JOS106	eNB(Y37am)::HL::mKate::His	JOS104 was amplified using primers to introduce an amber stop codon mutation at position 37.<br>J151 - Ecoli enhancer Y37am F: GATGAGATGGTAGCGGCAGGCGC<br>J152 - Ecoli enhancer Y37am R: GCGCCTGCCGCTACCATCTCATC
JOS107	eNB(Y37am, R35A)::HL::mKate::His	JOS106 was amplified using primers to introduce R35A mutation.<br>J258 - v3 Ecoli enhancer(Y37am) R35A F: TTCGATGGCGTGGTAGCGGCAGGCGCCC<br>J259 - v3 Ecoli enhancer(Y37am) R35A R: GCTACCACGCCATCGAATACCGGTTGACTGGGAACCCACTTGC
JOS115	eNB(Y37am, E103A)::HL::mKate::His	JOS106 was amplified using primers to introduce E103A mutation.<br>J161 - Ecoli enhancer E103A F: TGTCGGTTTTGCGTATTGGGGCC<br>J162 - Ecoli enhancer E103A R: GGCCCCAATACGCAAAACCGACA
SE383	pSANG pelB::eNB::HL::mKate::His	pSANG10-3F was digested with NcoI and EagI. eNB::HL::mKate was amplified from JOS104 using Seb primers 762 and 764. The fragments were assembled using NEBuilder.<br>Seb 762 eNB pSANG F: gctcctcgctgcccagccggccatggcccagATGGTTCAGCTGGTTGAGTC<br>Seb 764 eNB Ec pSANG R: atggtgatgatgatgtgcggatgcggccgcGGATCCAGATGAAACAGTGAC
SE391	pSANG pelB::eNB(E103A)::HL::mKate::His	pSANG10-3F was digested with NcoI and EagI. eNB(E103A)::HL::mKate was amplified from JOS168 using Seb primers 762 and 764. The fragments were assembled using NEBuilder.
SE387	pSANG pelB::eNB(R35A)::HL::mKate::His	pSANG10-3F was digested with NcoI and EagI. eNB(R35A)::HL::mKate was amplified from JOS170 using Seb primers 762 and 764. The fragments were assembled using NEBuilder.
SE385	pSANG pelB::eNB(Y37am)::HL::mKate::His	pSANG10-3F was digested with NcoI and EagI. eNB(Y37am)::HL::mKate was amplified from JOS106 using Seb primers 762 and 764. The fragments were assembled using NEBuilder.
SE393	pSANG pelB::eNB(Y37am, R35A)::HL::mKate::His	pSANG10-3F was digested with NcoI and EagI. eNB(Y37am, R35A)::HL::mKate was amplified from JOS107 using Seb primers 762 and 764. The fragments were assembled using NEBuilder.
SE389	pSANG pelB::eNB(Y37am, E103A)::HL::mKate::His	pSANG10-3F was digested with NcoI and EagI. eNB(Y37am, E103A)::HL::mKate was amplified from JOS115 using Seb primers 762 and 764. The fragments were assembled using NEBuilder.

A B

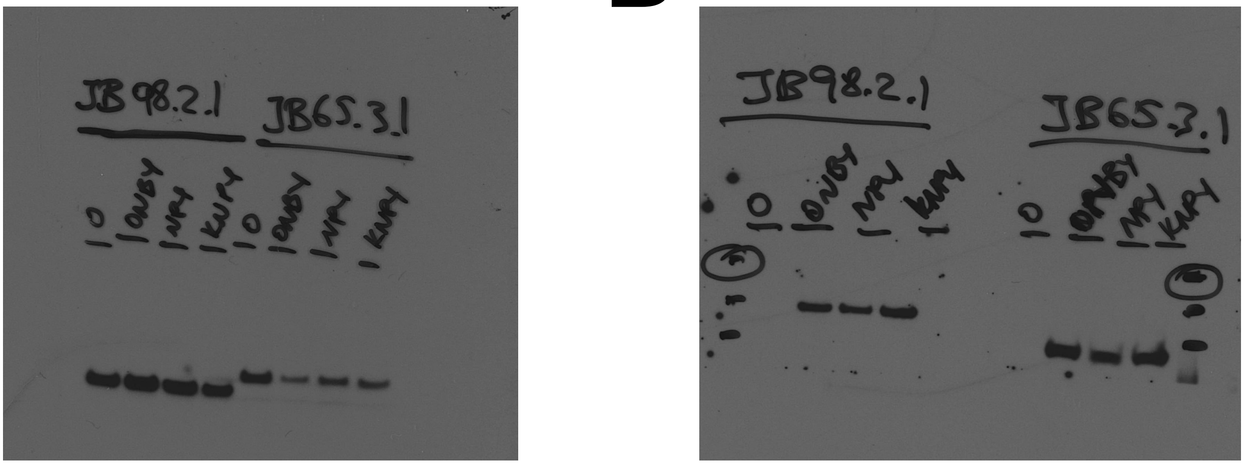

Supplementary Figure 1 Full Western blots of JB98 (SGR57) and JB65 (SGR58). **A.** Anti-GFP Western blots performed on lysates of strains JB98.2.1 (SGR57) and JB65.3.1 (SGR58) where animals were grown in the absence of non-canonical amino acid or in the presence of 0.1mM ONBY, NPY, or K-NPY **B. Left:** Anti-HA Western blots performed on lysates of strain JB98.2.1 (SGR57) where animals were grown in the absence of non-canonical amino acid or in the presence of 0.1mM ONBY, NPY, or K-NPY. **Right:** Anti-mCherry Western blots performed on lysates of strain JB65.3.1 (SGR58) where animals were grown in the absence of non-canonical amino acid or in the presence of 0.1mM ONBY, NPY, or K-NPY

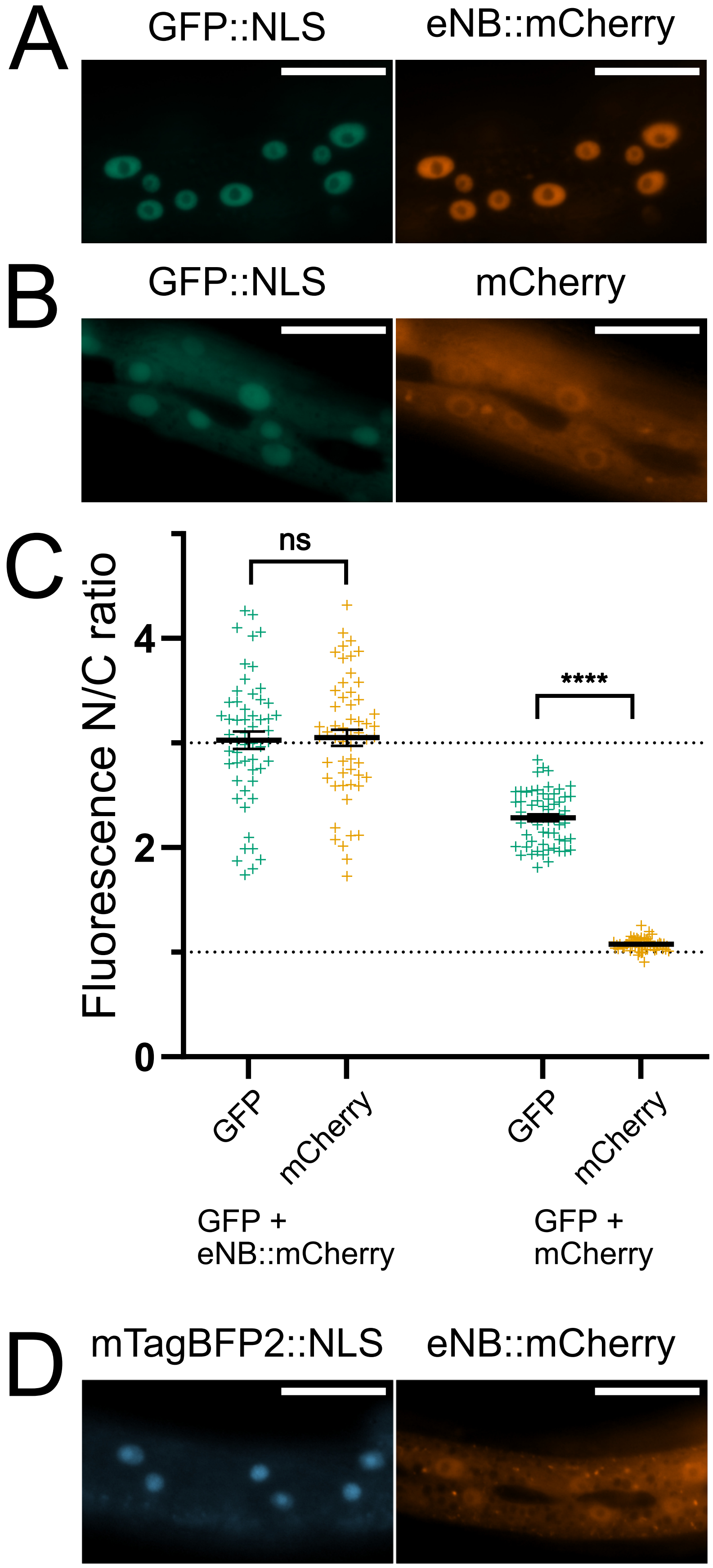

Supplementary Figure 2 A. Pictures of a *C.elegans* strain expressing wildtype eNB fused with mCherry and nuclear GFP. Scale bars of 20µm. **B.** Pictures of a *C.elegans* strain expressing mCherry and nuclear GFP. Scale bars of 20µm. **C.** Quantification of fluorescence nuclear/cytoplasmic ratio for strains shown in **A** and **B**. When GFP and eNB::mCherry are coexpressed, mean N/C ratio for GFP = 3.03 and for mCherry = 3.05. When GFP and mCherry are coexpressed, mean N/C ratio for GFP = 2.28 and for mCherry = 1.08. Data are presented as measurements of individual cells and mean ± SEM. Measurements were taken from 7-10 animals per condition. ns $p > 0.05$; **** $p < 0.0001$. **D.** Pictures of a *C.elegans* strain expressing

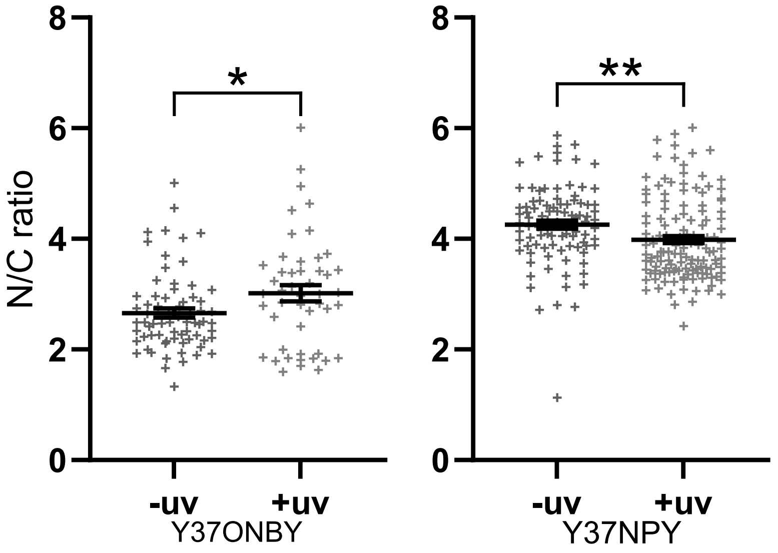

Supplementary Figure 3 Quantification of mCherry nuclear/cytoplasmic ratio for eNB with mutations Y37ONBY or Y37NPY. Data are presented as measurements of individual cells and mean ± SEM. Measurements were taken from 7-10 animals per condition.

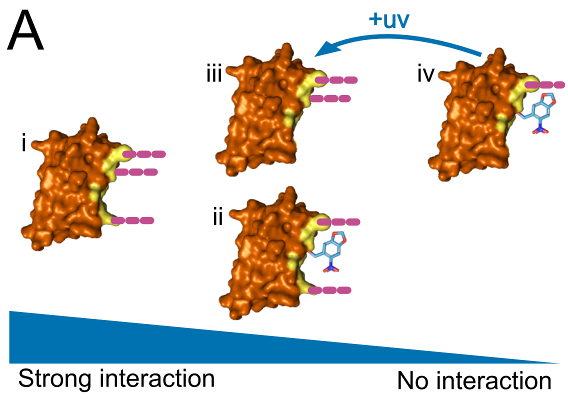

Supplementary Figure 4. Schematic for the design of an improved photocaged nanobody. The native nanobody **(i)** makes several interactions with its antigen. Photocaging **(ii)** or mutating **(iii)** one of these interactions is insufficient to abolish the interaction. Introducing a photocaging group and a mutation to the nanobody abolishes the interaction **(iv).** The interaction can be restored by removing the caging group through illumination with 365nm.

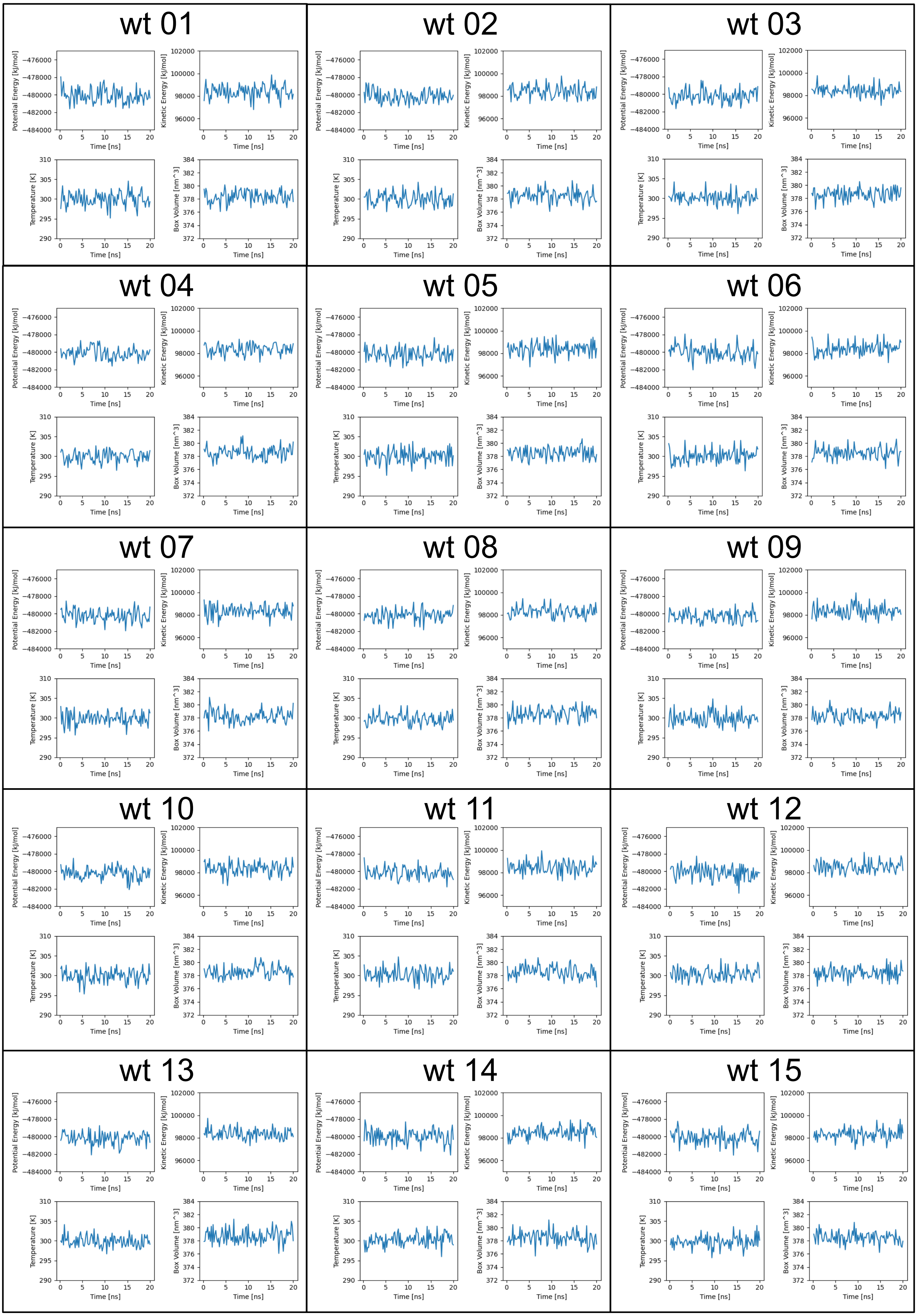

Supplementary Figure 5 Plots of potential energy, kinetic energy, temperature, and box volume of molecular-dynamics simulations for eNB^{wt}, eNB^{Y37ONBY}, and eNB^{Y37NPY}.

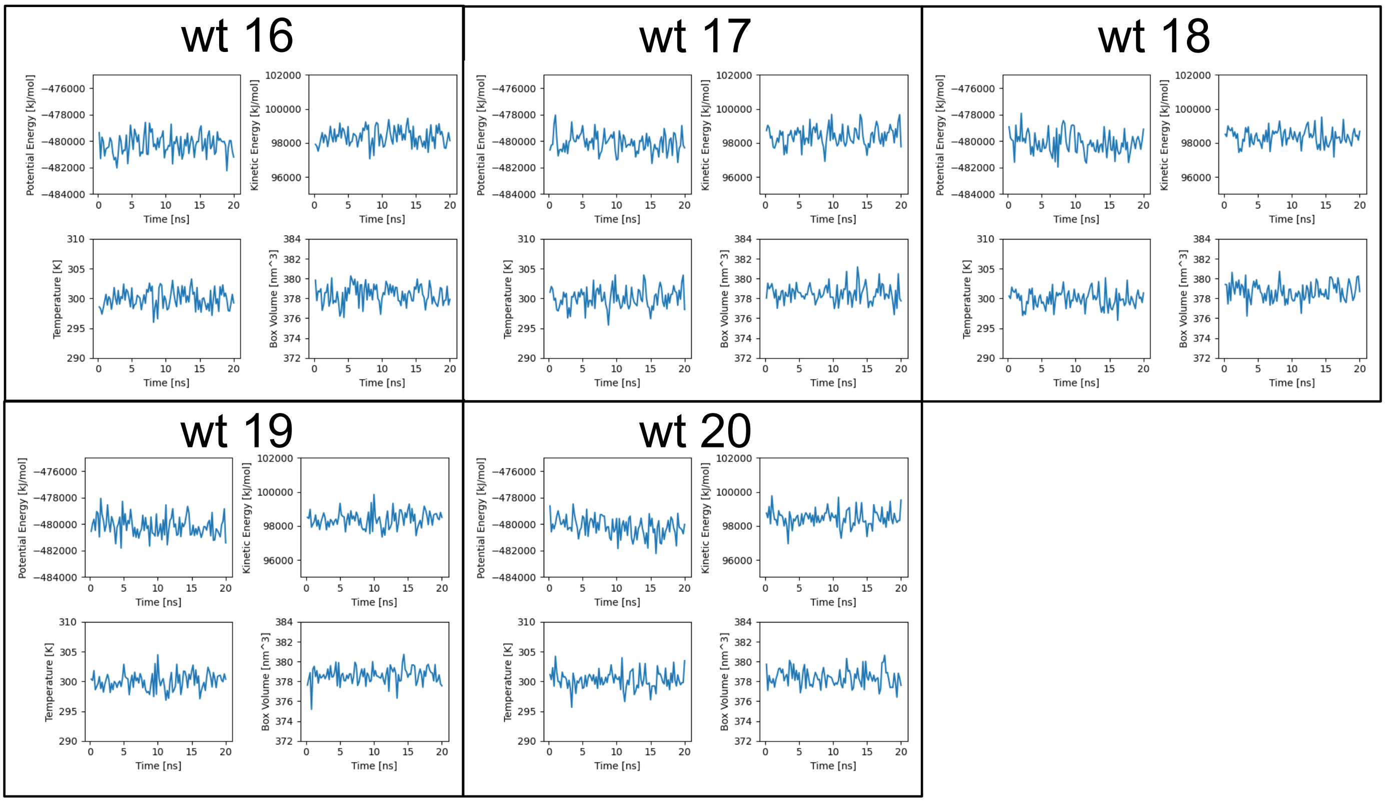

Supplementary Figure 5 continued.

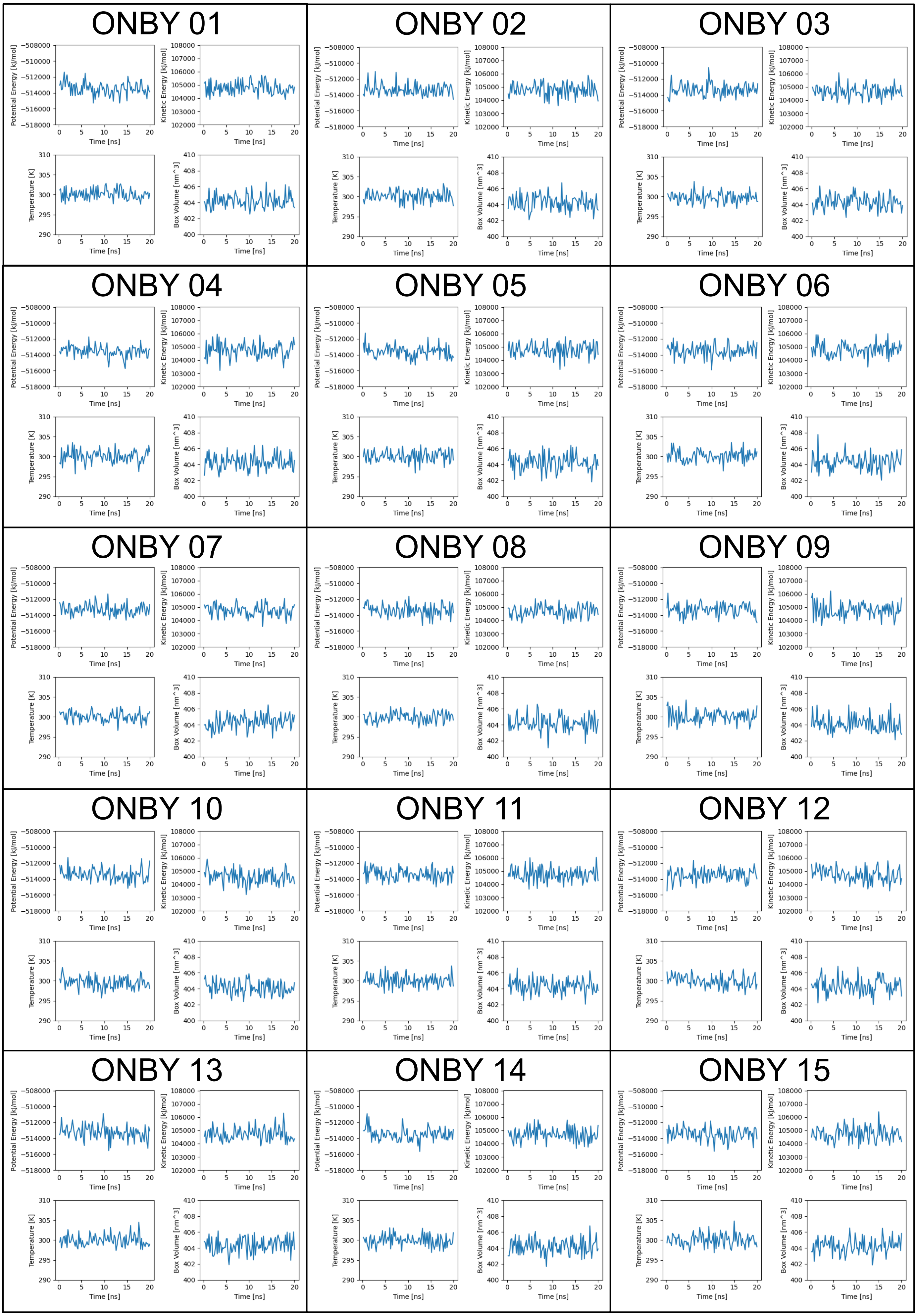

Supplementary Figure 5 continued.

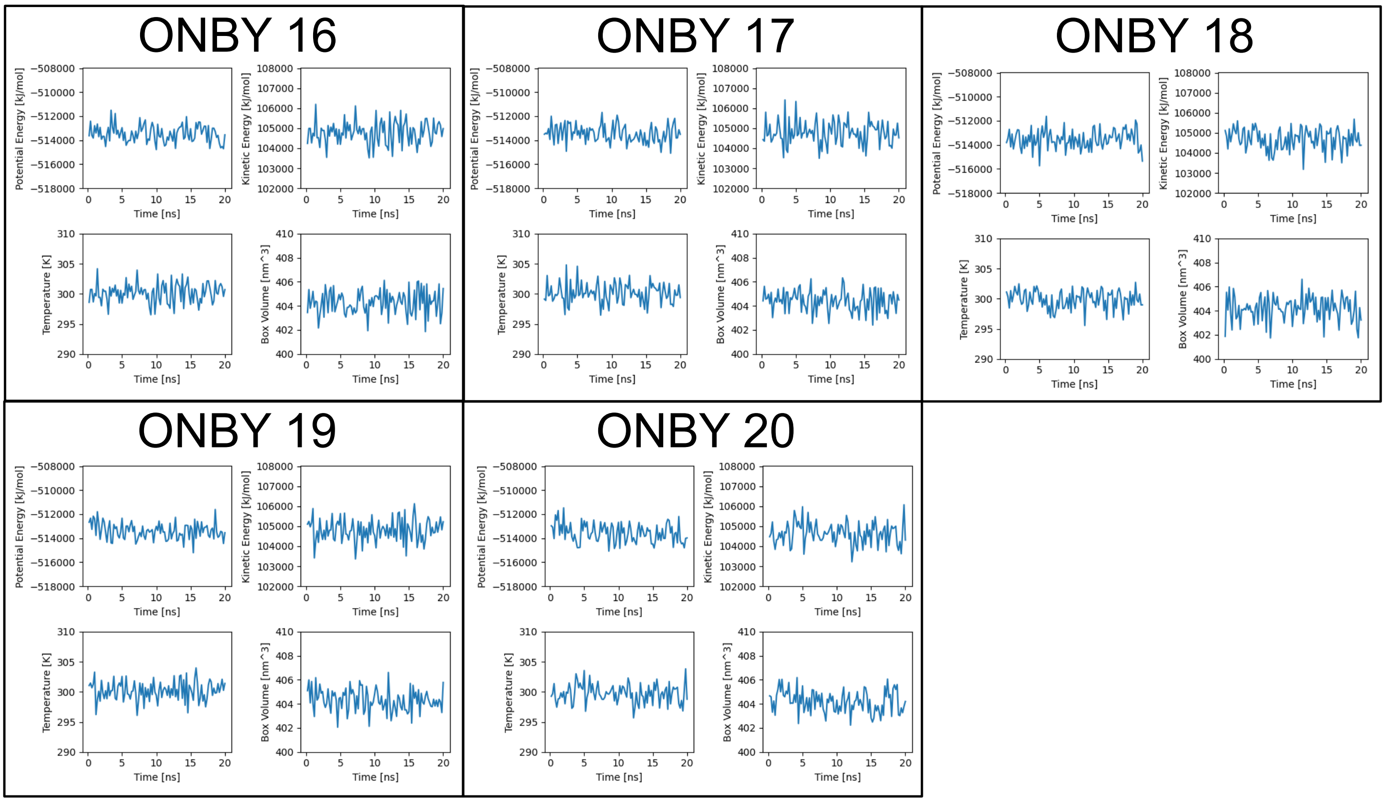

Supplementary Figure 5 continued.

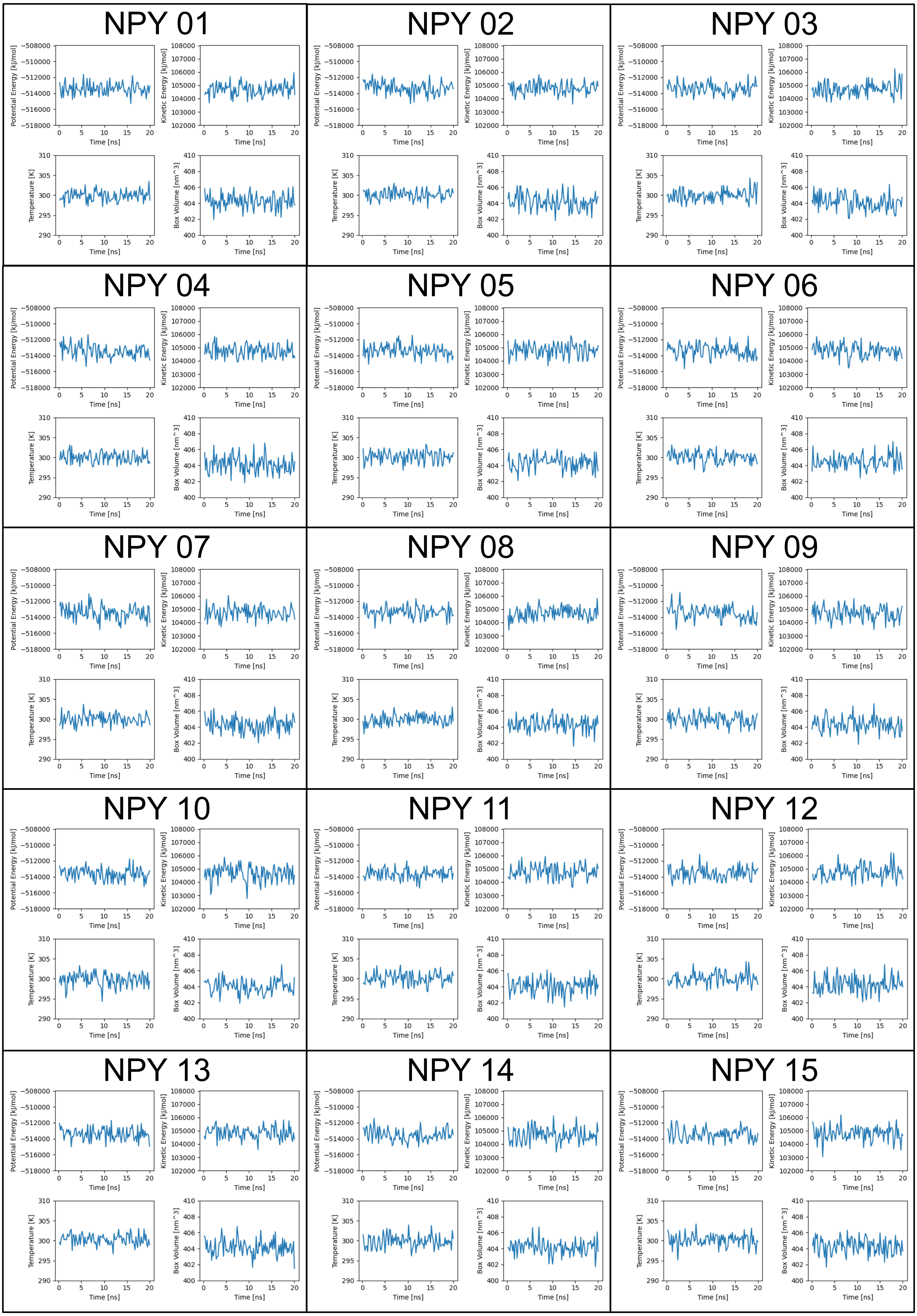

Supplementary Figure 5 continued.

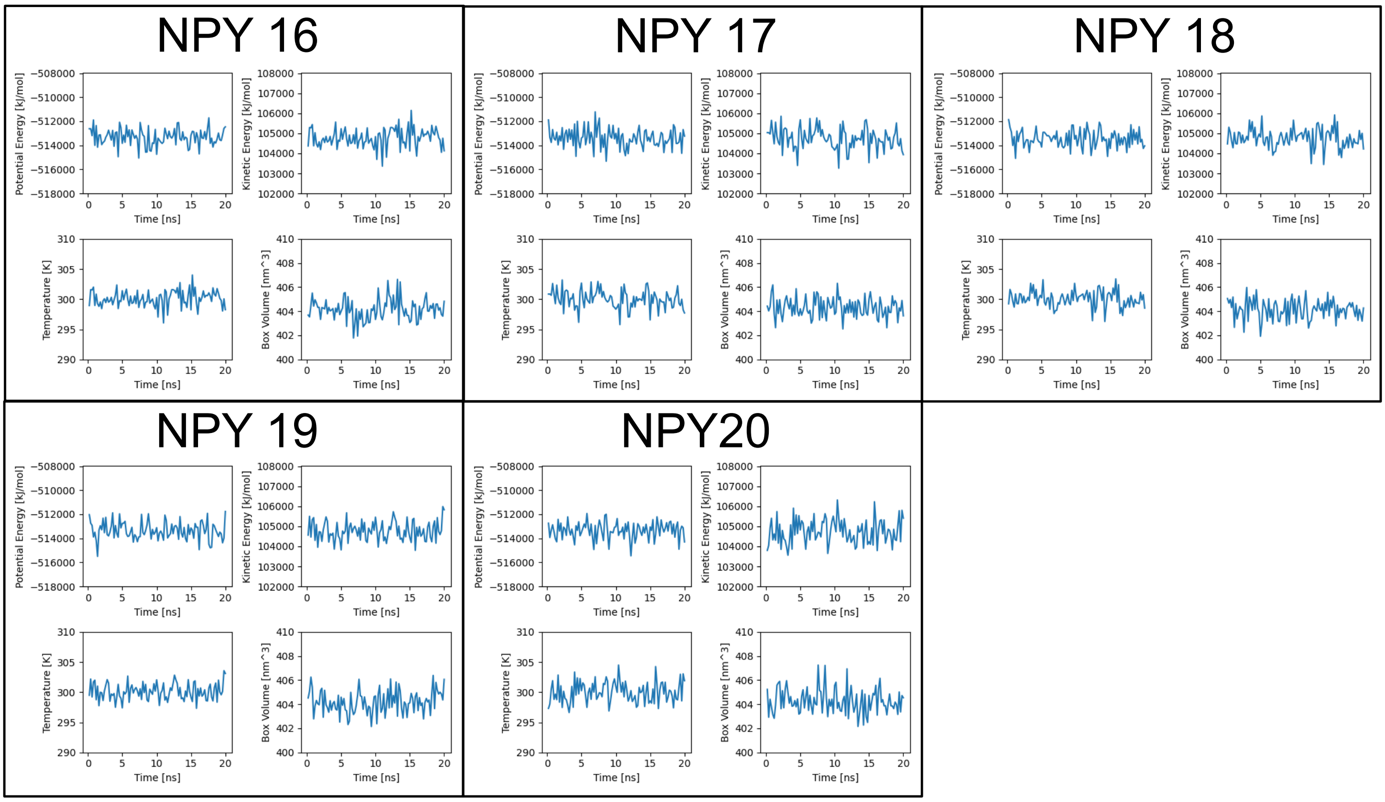

Supplementary Figure 5 continued.

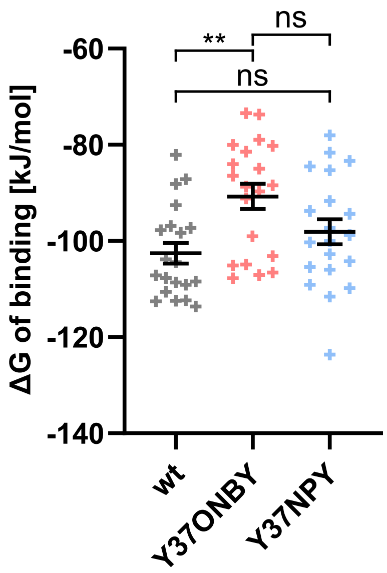

Supplementary Figure 6. BUDE Alanine Scan (BAlaS) calculations of ΔG of binding for the interaction between GFP and eNB^{wt}, eNB^{Y37ONBY}, or eNB^{Y37NPY}. The eNB^{wt}-GFP interaction has ΔG of -102.60kJ/mol (± 2.07). The eNB^{Y37ONBY}-GFP interaction has ΔG of -90.73kJ/mol (± 2.54). The eNB^{Y37NPY}-GFP interaction has ΔG of -98.09kJ/mol (± 2.54). Data are presented as individual simulations and mean ± SEM. ns $p > 0.05$; ** $p < 0.01$.

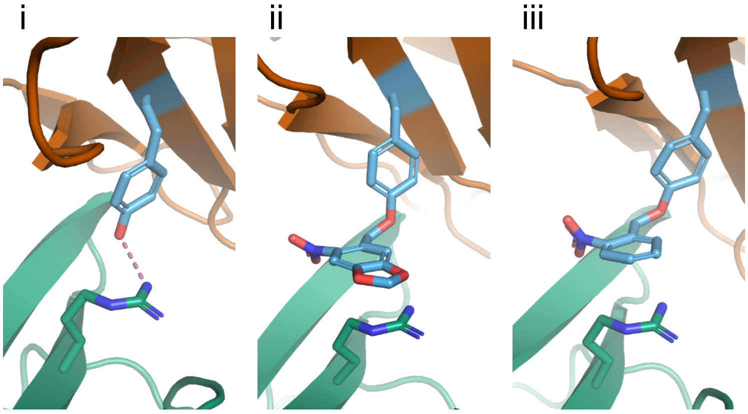

Supplementary Figure 7. Representative frames from MD trajectories for the interaction between R168 of GFP and Y37 **(i)**, NPY37**(ii)**, or ONBY37**(iii)** of eNB. The caging group of NPY and ONBY orient parallel to the GFP's R168 throughout molecular dynamic trajectories of the eNB-GFP complex, suggesting they could have an energetically favourable interaction.

A

Residue number	Residue name	ΔΔG (kJ/mol)
31	ARG	0.01
32	TYR	0.30
33	SER	1.53
34	MET	0.02
35	ARG	6.69
37	TYR	4.88
44	GLU	2.16
45	ARG	0.51
46	GLU	1.59
47	TRP	9.01
51	MET	0.21
52	SER	0.25
53	SER	0.43
56	ASP	0.41
57	ARG	3.67
59	SER	2.51
60	TYR	0.17
61	GLU	1.18
62	ASP	0.80
70	ILE	0.19
97	ASN	0.51
98	VAL	0.04
99	ASN	3.85
100	VAL	0.04
102	PHE	9.62
103	GLU	6.28
104	TYR	0.42
105	TRP	3.89

B

Residue number	Residue name	ΔΔG (kJ/mol)
102	PHE	9.62
47	TRP	9.01
35	ARG	6.69
103	GLU	6.28
37	TYR	4.88
105	TRP	3.89
99	ASN	3.85
57	ARG	3.67
59	SER	2.51
44	GLU	2.16
46	GLU	1.59
33	SER	1.53
61	GLU	1.18
62	ASP	0.80
45	ARG	0.51
97	ASN	0.51
53	SER	0.43
104	TYR	0.42
56	ASP	0.41
32	TYR	0.30
52	SER	0.25
51	MET	0.21
70	ILE	0.19
60	TYR	0.17
98	VAL	0.04
100	VAL	0.04
34	MET	0.02
31	ARG	0.01

C

```
  1 QVQLVESGGALVQPGGSLRLSCAASGFPVNRYSMRWYRQAPGKEREWVAG
 51 MSSAGDRSSYEDSVKGRFTISRDDARNTVYLQMNSLKPEDTAVYYCNVNV
101 GFEYWGQGTQVTVS
```

Supplementary Figure 8 A. Results of BUDE Alanine Scan analysis of eNB/GFP interaction ordered by residue number. The number and name of eNB residues are given along with the change in ΔG (ΔΔG) that occurs when the residue is mutated to alanine with respect to the wild-type ΔG. Residues selected for experimental analysis are highlighted in blue. The tyrosine selected for photocaging is higlighted in red. Ordered by residue number. **B.** Results of BUDE Alanine Scan analysis of eNB/GFP interaction ordered by ΔΔG. **C.** Amino acid sequence of eNB.

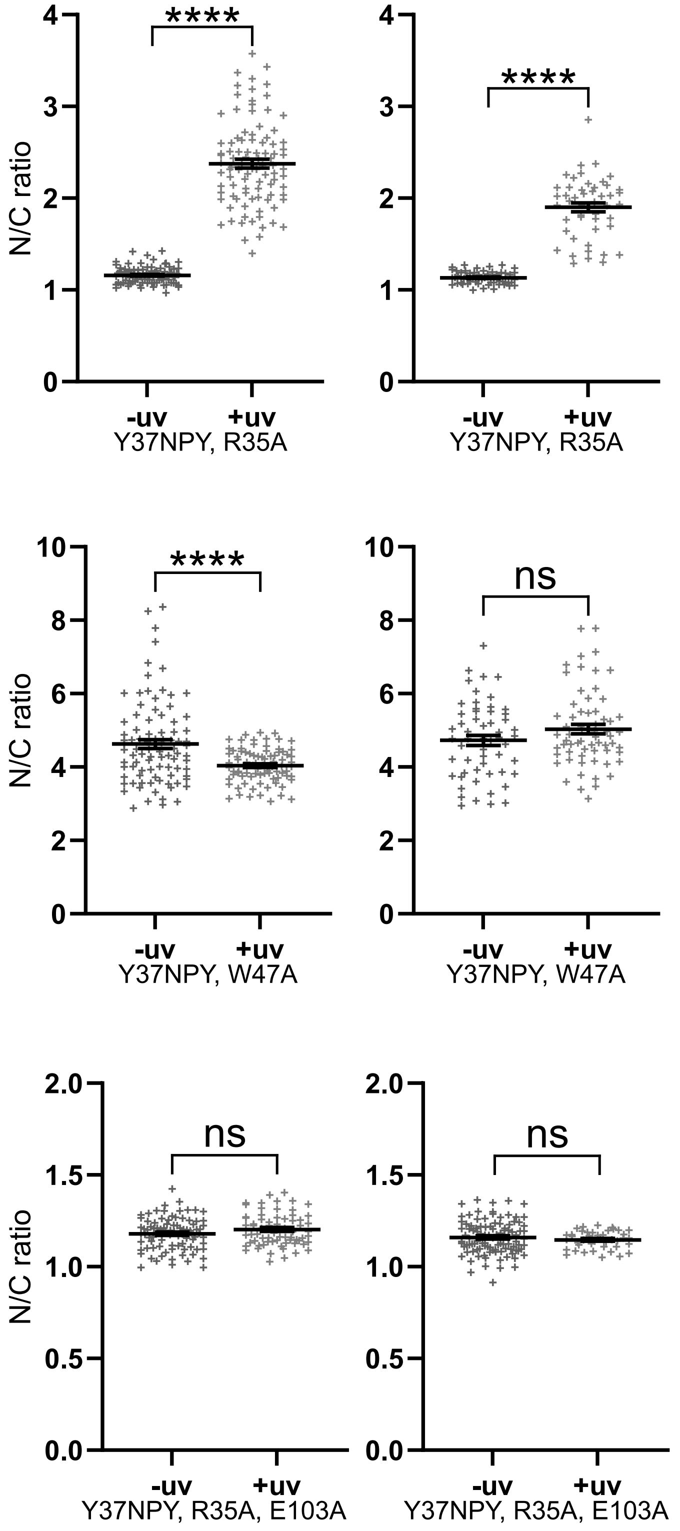

Supplementary Figure 9. Repeat quantifications of mCherry nuclear/cytoplasmic ratio for eNB variants. Data are presented as measurements of individual cells and mean ± SEM. Measurements were taken from 7-10 animals per condition.

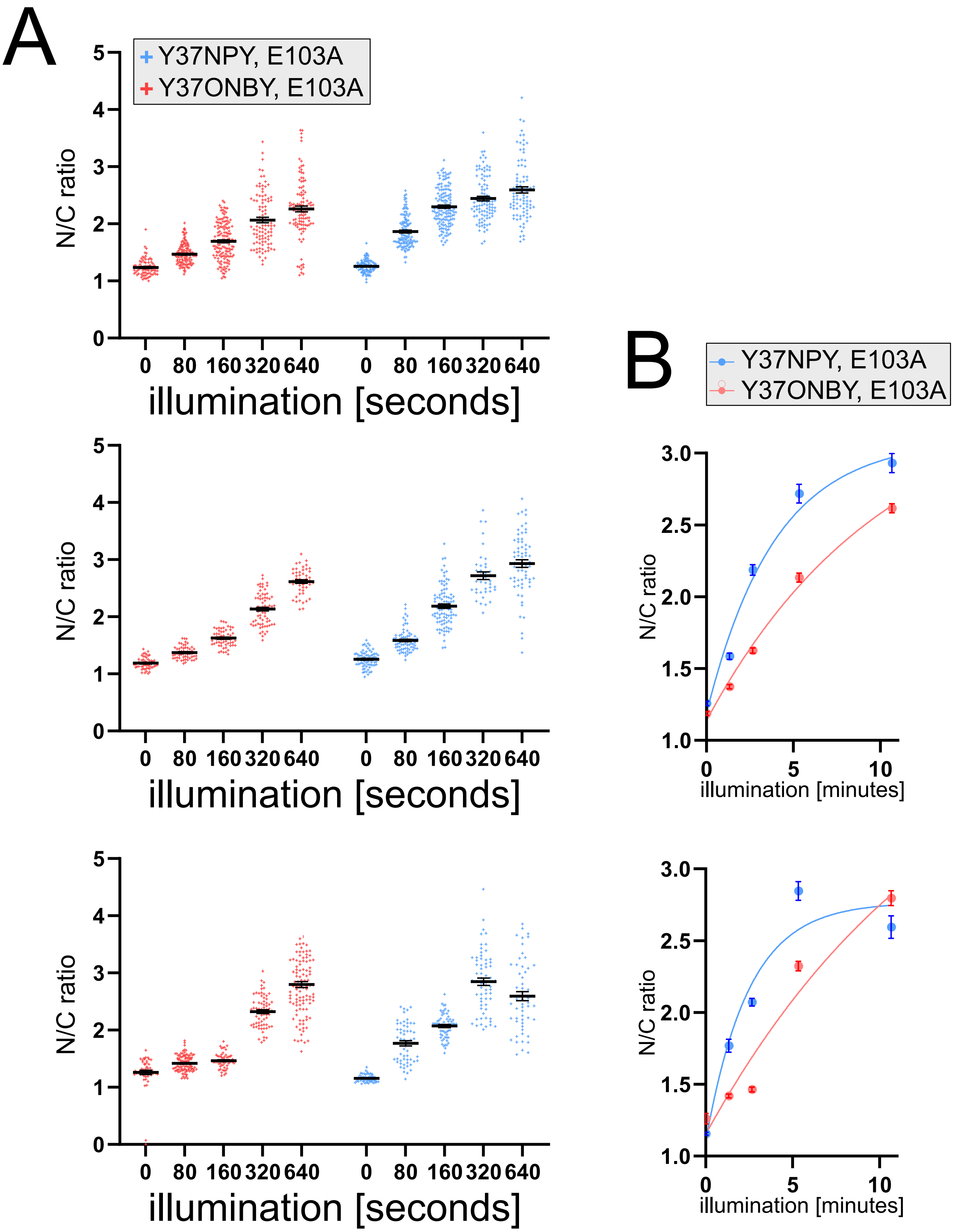

Supplementary Figure 10. A. Quantification of mCherry nuclear/cytoplasmic ratio of eNB$^{Y37ONBY, E103A}$ and eNB$^{Y37NPY, E103A}$ subjected to a range of 365nm illumination times. Data are presented as individual cell measurements and mean ± SEM. Measurements were taken from 7-10 animals per condition. **B.** Quantification of mCherry nuclear/cytoplasmic ratio of eNB$^{Y37ONBY, E103A}$ and eNB$^{Y37NPY, E103A}$ subjected to a range of 365nm illumination times. Data are presented as mean ± SEM. Measurements were taken from 7-10 animals per condition.

A

Residue number	Residue name	ΔΔG (kJ/mol)
37	PHE	0.15
43	LYS	0.02
44	GLU	0.58
46	GLU	0.13
47	LEU	0.32
50	ASN	0.05
52	LEU	0.08
58	THR	0.19
64	LYS	0.60
98	ASP	6.88
101	THR	3.97
103	LEU	7.63
105	TYR	17.90
106	VAL	0.74
109	VAL	1.65
111	LEU	0.42
115	ASP	0.68
116	TYR	9.85
117	VAL	3.71
118	MET	0.11
119	ASP	6.35
120	TYR	0.18
121	TRP	0.03

B

Residue number	Residue name	ΔΔG (kJ/mol)
105	TYR	17.90
116	TYR	9.85
103	LEU	7.63
98	ASP	6.88
119	ASP	6.35
101	THR	3.97
117	VAL	3.71
109	VAL	1.65
106	VAL	0.74
115	ASP	0.68
64	LYS	0.60
44	GLU	0.58
111	LEU	0.42
47	LEU	0.32
58	THR	0.19
120	TYR	0.18
37	PHE	0.15
46	GLU	0.13
118	MET	0.11
52	LEU	0.08
50	ASN	0.05
121	TRP	0.03
43	LYS	0.02

C

```
  1 DVQLQESGGGSVQAGGSLRLSCAASGDTFSSYSMAWFRQAPGKECELVSN
 51 ILRDGTTTYAGSVKGRFTISRDDAKNTVYLQMVNLKSEDTARYYCAADSG
101 TQLGYVGAVGLSCLDYVMDYWGKGTQVTVS
```

Supplementary Figure 11 A. Results of BUDE Alanine Scan analysis of mNB/GFP interaction ordered by residue number. The number and name of mNB residues are given along with the change in ΔG (ΔΔG) that occurs when the residue is mutated to alanine with respect to the wild-type ΔG. Residues selected for experimental analysis are highlighted in blue. The tyrosine selected for photocaging is higlighted in red. Ordered by residue number. **B.** Results of BUDE Alanine Scan analysis of mNB/GFP interaction ordered by ΔΔG. **C.** Amino acid sequence of mNB.

References

[1] S. Brenner, *Genetics.* **1974**, *77*, 71–94.

[2] T. Stiernagle, *C. elegans* **1999**, *2*, 51–67.

[3] S. Redemann, S. Schloissnig, S. Ernst, A. Pozniakowsky, S. Ayloo, A. A. Hyman, H. Bringmann, *Nat. methods.* **2011**, *8*, 250–252.

[4] I. Radman, S. Greiss, J. W. Chin, *PLoS One* **2013**, *8*, e76019.

[5] J. B. Kelley, B. M. Paschal, *Methods* **2019**, *157*, 106–114.

[6] D. A. Case, I. Y. Ben-Shalom, S. R. Brozell, D. S. Cerutti, T. E. Cheatham III, T. A. Cruzeiro, V.W.D., Darden, R. E. Duke, D. Ghoreishi, M. K. Gilson, H. Gohlke, A. W. Goetz, D. Greene, R. Harris, N. Homeyer, Y. Huang, S. Izadi, A. Kovalenko, T. Kurtzman, T. S. Lee, S. LeGrand, P. Li, C. Lin, J. Liu, T. Luchki, R. Luo, D. J. Mermelstein, K. M. Merz, Y. Miao, G. Monard, C. Nguyen, H. Nguyen, I. Omelyan, A. Onufriev, F. Pan, R. Qi, D. R. Roe, A. Roitberg, C. Sagui, S. Schott-Verdugo, J. Shen, C. L. Simmerling, J. Smith, F. Salomon-Ferrer, J. Swails, R. C. Walker, J. Wang, H. Wei, R. M. Wolf, X. Wu, L. Xiao, D. M. York, P. A. Kollman, *Univ. California, San Fr.* **2018**.

[7] Y. Myung, S. H. Han, *Bull. Korean Chem. Soc* **2010**, *31*, 2581–2587.

[8] N. M. O’Boyle, M. Banck, C. A. James, C. Morley, T. Vandermeersch, G. R. Hutchison, *J. Cheminform.* **2011**, *3*, 1–14.

[9] P. Eastman, J. Swails, J. D. Chodera, R. T. McGibbon, Y. Zhao, K. A. Beauchamp, L.-P. Wang, A. C. Simmonett, M. P. Harrigan, C. D. Stern, R. P. Wiewoira, B. R. Brooks, V. S. Pande, *PLoS Comput. Biol.* **2017**, *13*, e1005659.

[10] S. Greiss, J. W. Chin, *J. Am. Chem. Soc.* **2011**, *2*, 14196–14199.

[11] L. Davis, I. Radman, A. Goutou, A. Tynan, K. Baxter, Z. Xi, J. W. Chin, S. Greiss, *bioRxiv* **2020**, DOI 10.1101/2020.05.02.072363.

[12] C. D. Martin, G. Rojas, J. N. Mitchell, K. J. Vincent, J. Wu, J. McCafferty, D. J. Schofield, *BMC Biotechnol.* **2006**, *6*, 1–15.

[13] K. C. Schultz, L. Supekova, Y. Ryu, J. Xie, R. Perera, P. G. Schultz, *J. Am. Chem. Soc.* **2006**, *128*, 13984–13985.
